# Accuracy and Effectiveness of Mammography versus Mammography and Tomosynthesis for Population-Based Breast Cancer Screening: A Systematic Review and Meta-Analysis

**DOI:** 10.1038/s41598-020-64802-x

**Published:** 2020-05-14

**Authors:** Rodrigo Rosa Giampietro, Marcos Vinicius Gama Cabral, Silvana Andrea Molina Lima, Silke Anna Theresa Weber, Vania dos Santos Nunes-Nogueira

**Affiliations:** 1 Department of Internal Medicine, São Paulo State University/UNESP, Medical School, Botucatu, Sao Paulo Brazil; 2 Department of Nursing, São Paulo State University/UNESP, Medical School, Botucatu, Sao Paulo Brazil; 3 Health Technology Assessment Nucleus, Botucatu Medical School Clinical Hospital, Sao Paulo, Brazil; 4 Ophthalmology, Otorhinolaryngology and Head & Neck Surgery Department, São Paulo State University/UNESP, Medical School, Botucatu, Sao Paulo Brazil

**Keywords:** Cancer imaging, Cancer imaging

## Abstract

We proposed to compare the accuracy and effectiveness of digital breast tomosynthesis (DBT), plus digital or synthetic mammography, with digital mammography alone in women attending population-based breast cancer screenings. We performed a systematic review and included controlled studies comparing DBT with digital mammography for breast cancer screening. Search strategies were applied to the MEDLINE, Embase, LILACS, and CENTRAL databases. With moderate quality of evidence, in 1,000 screens, DBT plus digital mammography increased the overall and invasive breast cancer rates by 3 and 2 (RR 1.36, 95% CI 1.18 to 1.58 and RR 1.51, 95% CI 1.27 to 1.79, respectively). DBT plus synthetic mammography increased both overall and invasive breast cancer rates by 2 (RR 1.38, 95% CI 1.24 to 1.54 and RR 1.37, 95% CI 1.22 to 1.55, respectively*)*. DBT did not improve recall, false positive and false negative rates. However due to heterogeneity the quality of evidence was low. For women attending population-based breast cancer screenings, DBT increases rates of overall and invasive breast cancer. There is no evidence with high or moderate quality showing that DBT compared with digital mammography decreases recall rates, as well as false positive and false negative rates.

## Introduction

Breast cancer is one of the most frequently diagnosed cancers among women, and population-based breast cancer screenings with mammography have been one of the worldwide health strategies to reduce breast cancer mortality^1^.

Technological advances in image acquisition provided the transition from film screen to digital mammography. In more recent years, as an advancement from mammography, digital breast tomosynthesis (DBT) has been introduced into screening practices which has the potential to overcome limitations of digital mammography^2^. Most diagnostic centres perform DBT with digital mammography. However some software can synthesize digital mammography images (synthetic mammography) from data acquired during DBT, thus reducing the radiation dose^3^.

Several studies have shown that adding DBT to digital mammography significantly increases the detection of breast cancer^4–6^. However, results from previous studies regarding recall rates are inconsistent; some studies have shown reduction in false recalls^7,8^, while others have shown that the proportion of women recalled for further assessment has increased^9,10^.

Although there are several systematic reviews on this topic in the literature, none have included DBT with synthetic mammography in their analysis^11–23^. In addition, at least three clinical trials evaluating the effectiveness of DBT on breast cancer screening have been reported since these reviews were published^24–26^.

Thus, we proposed to evaluate the accuracy and effectiveness of DBT (with digital mammography or synthetic mammography) compared to digital mammography alone in women with a standard risk for developing this neoplasia, who attended population-based breast cancer screenings.

## Methods

A systematic review was conducted according to the Cochrane Handbook for Systematic Reviews of Diagnostic Test Accuracy^27^ and was reported on according to the PRISMA- Diagnostic Test Accuracy Studies (DTAs) Statements^28,29^. Our protocol was registered in the International Prospective Registry of Systematic Reviews, under the ID, CRD42017070890.

### Eligibility criteria

#### Type of studies

We included randomized (RCT) and quasi-randomized controlled trials (quasi-RCT), cohort studies, and diagnostic test accuracy studies (cross-sectional studies involving patients who received mammography and DBT, and in which screen-reading was performed in two sequential phases, mammography only versus mammography integrated with DBT). The included studies followed the PICO protocol described below:

#### Patients (P)

We included studies involving women, over 45 years of age and with no breast cancer related symptoms, from among a population with a standard risk of developing breast cancer, who attended population-based breast cancer screenings.

#### Index test (I)

We considered DBT, either with digital mammography or synthetic mammography, as the index test.

#### Comparison (C)

We considered digital mammography alone as the comparison test.

#### Types of outcome measures (O) of the included studies

Primary outcomes were overall and breast cancer mortalities, overall invasive and non-invasive breast cancer detection rates, proportion of women recalled for additional examinations (recall rate), adverse events, and irradiation dose per examination.

Secondary outcomes were the true positive, false positive, false negative, and true negative rates. If such data were available, the accuracy of each index test was calculated (sensitivity, specificity, positive and negative predictive values, positive and negative likelihood ratios, and diagnostic odds ratios).

#### Reference test

As a reference test to confirm the positive cases of breast cancer, we considered the results of histological tests conducted after surgery or by biopsy. To confirm the negative cases, we considered the absence of breast cancer detected via examinations during a follow-up period.

#### Exclusion criteria

We excluded studies in which participants consisted of women with established risk factors for breast cancer, and studies in which most participants were already diagnosed with some type breast disease or were called for additional examinations.

Further, we excluded studies in which the index and comparator tests were performed at different times.

For studies that met the eligibility criteria but also included women who were under 45 years old, an e-mail was sent to the corresponding author requesting the outcome data for patients over 45 years old. Studies that did not provide this information were included if most of the sample comprised of women aged according to our eligibility criteria.

#### Search methods for identification of studies

Four general and adaptive search strategies were created for the electronic databases: Embase (1980-01/March/2020), PubMed (1966-01/March/2020), LILACS (1982-01/March/2020), and CENTRAL (Cochrane Collaboration Controlled Trials Registry-01/March/2020) (Supplementary File). The mesh terms—breast cancer and DBT— were used to construct each search strategy; there were no language or year restrictions (Supplementary File).

Additionally, we surveyed the Trip Medical Database, SCOPUS, Web of Science, and CINAHL. Furthermore, we searched thesis banks for unpublished studies and ClinicalTrials.gov for ongoing studies.

We used the Endnote software to download references, remove duplicates, and facilitate the selection process.

#### Selection of studies

Two reviewers independently (VSNN and RRG) selected titles and abstracts from the ones identified via the bibliographic research. Potentially eligible studies were selected for a full reading and, subsequently, evaluated for conformance to the proposed PICO. In case of disagreements during the selection process, we arrived at a consensus via discussions. The reasons behind each excluded study were justified.

#### Data extraction and management

Both reviewers applied a data extraction form to the studies to compute the corresponding participant-related information.

#### Risk of bias and applicability

We evaluated the risk of bias corresponding to the included studies via the QUADAS-2 (Quality Assessment of Diagnostic Accuracy Studies) tool^30^.

#### Unit of analysis

The unit of analysis was the aggregated data extracted from the journal publications.

#### Assessment of heterogeneity

Inconsistencies among the study results were ascertained by visually inspecting a forest plot and with the Higgins or I^2^ statistic, in which an I^2^ > 50% indicated a moderate probability of heterogeneity.

#### Synthesis of results (Meta-analysis)

Similar outcomes, measured in at least two trials, were plotted in the meta-analysis using Review Manager 5.3 (Review Manager. [RevMan], version 5.3, Copenhagen: The Nordic Cochrane Centre, The Cochrane Collaboration, 2014). For dichotomic outcomes, the relative risk (RR) was calculated with a 95% confidence interval (CI) as an effect size of the effectiveness of the index test. We selected the random effects model for the meta-analysis, and the studies were evaluated separately according to their designs.

#### Grading the quality of evidence

For each outcome, a tabulated summary of the findings was produced in order to report the effectiveness of the index test. The certainty of the evidence was measured using the GRADE approach (Grading of Recommendations Assessment, Development, and Evaluation Working Group)^31^.

### Ethical standards

As no primary data collection was undertaken, no formal ethical assessment is required in our institution.

## Results

### Study selection

**Figure 1:**
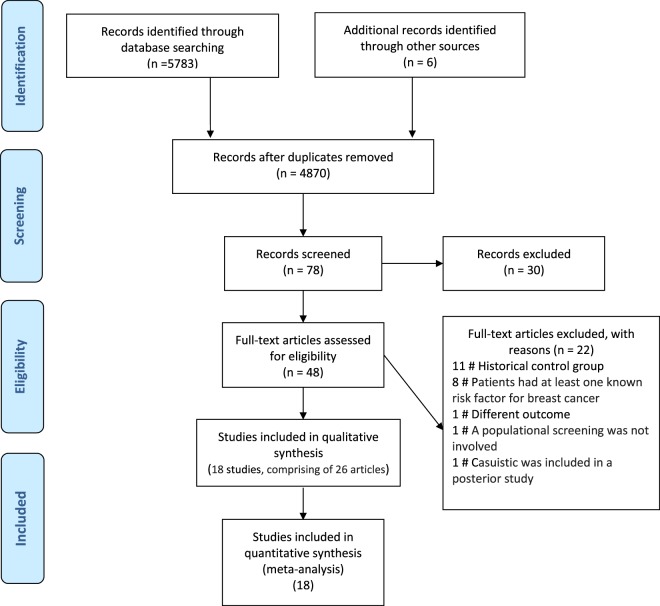
Flow of selection of articles for the systematic review.

The search strategies yielded 5,783 references, and after removing duplicates, 4,870 studies remained. We selected 48 studies that had a high probability of meeting our inclusion criteria for a complete reading (Fig. 1).

After completely reading these references, 18 studies (comprising 26 articles, since some had more than one published article) met our eligibility criteria and therefore were included in this review^4–8,24–26,32–49^.

A total of 22 studies were excluded for the following reasons: 11 used a historic cohort as control;^50–60^ eight studies included patients that had at least one known risk factor for breast cancer or were invited to perform additional tests due to alterations in previous examinations;^61–68^ in one study the outcome evaluated was interpretation time of screening examinations^69^, in one study a populational screening was not involved^70^, and in one study the patients included were also included in a posterior study^71^.

### Study characteristics

Only two trials explicitly include asymptomatic women at a standard population risk for breast cancer^7,35^. In the other studies, the eligibility criterion was women who attended population screening programs. Therefore, it is inferred that most participants were asymptomatic and at a population standard risk.

Fourteen studies evaluated DBT in combination with digital mammography versus digital mammography alone. These studies included: one RCT^24^, five accuracy studies^7,34,35,40,44^, and eight retrospective cohort studies^4–6,8,36–38,46^. Six studies, three accuracy studies^35,43,48^, one prospective cohort study^47^, one RCT^25^ and one quasi-RCT^26^ evaluated the effectiveness of DBT with synthetic mammography versus digital mammography alone.

In all included studies, the radiologists were experienced in breast imaging and had received trainings on DBT. Three studies had an eligibility criterion of including women older than 40 years, two of women older than 45 years, and the remaining included women older than 50.

Table X of supplementary data presents descriptive data of all the included studies.

### Risk of bias and applicability

**Figure 2:**
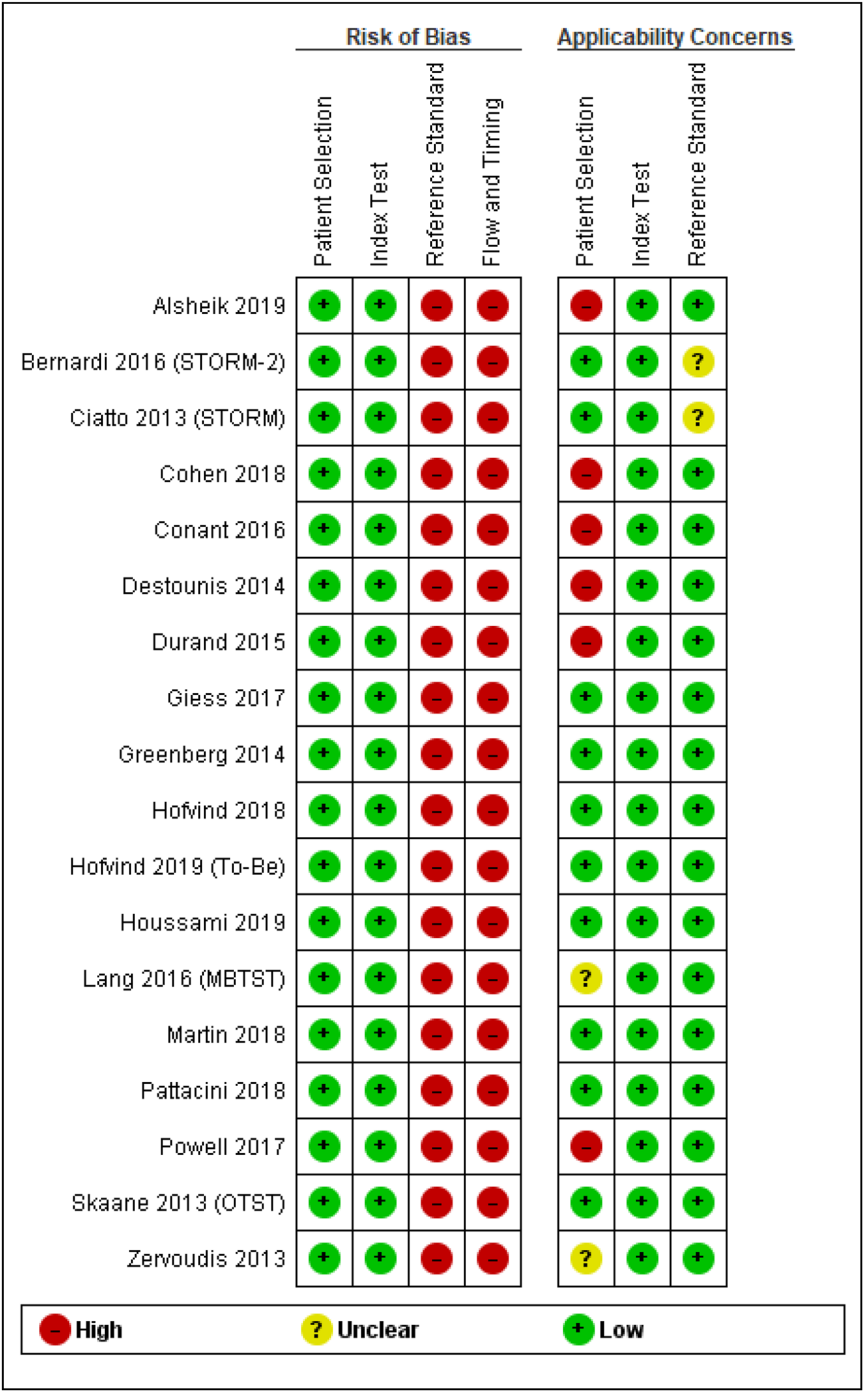
Risk of bias assessment according to QUADAS 2.

Figure 2 shows the risk of bias corresponding to the included studies. Most retrospective cohort studies were assessed as having a high probability of bias in patient selection (the DBT group had more risk factors for breast cancer). The studies involving patients under 45 years old were deemed to have an uncertain risk regarding the applicability of the patient selection. All studies were evaluated as having a high risk of bias in the reference test, since the pathologists who evaluated the biopsies and pathological results had prior knowledge of the screening tests. Follow-ups were also evaluated as having a high risk of bias, since patients who were not recalled missed the reference test.

### Meta-analysis DBT plus digital mammography versus digital mammography alone

#### Breast Cancer Detection Rate (Fig. 3 to Fig. 7, Supplementary File)

Based on RCT and accuracy studies, with a moderate quality of evidence and in 1,000 screened women, DBT plus digital mammography increased the overall breast cancer rates by 3 (RR 1.36, 95% CI 1.18 to 1.58, Table 1), and the rate of invasive breast cancer detection was increased by 2 (RR 1.51, 95% CI 1.27 to 1.79, Table 1). Regarding the rate of ductal carcinoma *in situ*, there was no clear evidence to support a difference between the two interventions (RR 1.26 95% CI 0.86 to 1.83).

**Table 1:** Summary of findings from the main comparisons.

**DBT plus either conventional digital mammography or synthetic mammography compared to conventional digital mammography alone in women attending population-based breast cancer screenings**						
**Patient:** Women attending population-based breast cancer screenings **Setting**: Population-based screening programs **Intervention**: DBT plus either conventional digital mammography or synthetic mammography **Comparison**: Conventional digital mammography alone						
Outcomes	Anticipated absolute effects^*^ (95% CI)		Relative effect (95% CI)	№ of participants (studies)	Certainty of the evidence (GRADE)	Comments
	**Risk with DM alone**	**Risk with DBT** + **DM or SM**				
Breast cancer detection rate from RCT and DAT studies - **DBT** + **DM**	7 per 1.000	**10 per 1.000**(8 to 11)	**RR 1.36**(1.18 to 1.58)	58265(1 RCT, 5 DTAs)	⨁⨁⨁◯MODERATE ^a^	**DBT plus DM** likely increases breast cancer detection rate slightly.
Invasive breast cancer detection rate from RCT and DAT studies- **DBT** + **DM**	5 per 1.000	**7 per 1.000**(6 to 8)	**RR 1.51**(1.27 to 1.79)	56650(1 RCT, 4 DTAs)	⨁⨁⨁◯MODERATE ^a^	**DBT plus DM** likely increases invasive breast cancer detection rate.
Recall Rate from RCT and DAT studies- **DBT** + **DM**	34 per 1.000	**38 per 1.000**(32 to 45)	**RR 1.13**(0.96 to 1.32)	58265(1 RCT, 5 DTAs)	⨁⨁◯◯LOW ^a, b, c^	**DBT** + **DM** may increase/have little to no effect on recall Rate DBT + DM but the evidence is very uncertain.
Breast Cancer Detection Rate - **DBT** + **SM**	6 per 1.000	**8 per 1.000**(7 to 9)	**RR 1.38**(1.24 to 1.54)	175572(2 RCT, 3 DTAs, 1 PC)	⨁⨁⨁◯MODERATE ^a^	**DBT** + **SM** likely increases breast cancer detection rate.
Recall Rate - **DBT** + **SM**	33 per 1.000	**35 per 1.000**(30 to 41)	**RR 1.08**(0.92 to 1.26)	175572(2 RCT, 3 DTAs, 1 PC)	⨁⨁◯◯LOW ^a, d^	**DBT** + **SM** may result in no difference in recall rate.
Invasive breast cancer detection rate - **DBT** + **SM**	5 per 1.000	**7 per 1.000**(6 to 8)	**RR 1.37**(1.22 to 1.55)	163604(2 RCT, 2 DTAs, 1 PC)	⨁⨁⨁◯MODERATE ^a^	**DBT** + **SM** likely increases the rate of invasive breast cancer.
*The risk in the intervention group (and its 95% confidence interval) is based on the assumed risk in the comparison group and the **relative effect** of the intervention (and its 95% CI).**DBT**: Digital breast tomosynthesis**; DM:** Digital Mammography**; SM:** Synthetic mammography**, RCT:** Randomized Clinical Trial; **DAT**: Diagnostic test accuracy study; **PC:** Prospective cohort**; CI:** Confidence interval; **RR:** Risk ratio						
**GRADE Working Group grades of evidenceHigh certainty:** We are very confident that the true effect lies close to that of the estimate of the effect**Moderate certainty:** We are moderately confident in the effect estimate: The true effect is likely to be close to the estimate of the effect, but there is a possibility that it is substantially different**Low certainty:** Our confidence in the effect estimate is limited: The true effect may be substantially different from the estimate of the effect**Very low certainty:** We have very little confidence in the effect estimate: The true effect is likely to be substantially different from the estimate of effect						

Explanations

a. The pathologists who evaluated the biopsies and pathological results had prior knowledge of the screening tests. Follow-ups were also evaluated as a high risk of bias, since patients who were not recalled missed the reference test

b. Wide confidence interval

c. In three studies DBT plus DM increased the recall rates, in two studies there was no difference between the groups

d. In three studies DBT plus SM did not show difference between the groups, in two studies it increased the recall rate, and in one study DBT plus SM decreased the recall rate.

Based on retrospective cohort studies, the rates of overall, invasive and ductal breast cancer are very similar to those of RCT and accuracy studies. However, the certainty of the evidence was lower due to the fact that women in the DBT group had more risk factors for breast cancer than those in the digital mammography alone group.

#### Recall Rate (Fig. 8 and Fig. 9, Supplementary File)

RCT and accuracy studies with DBT plus digital mammography did not reveal differences in recall rates compared to those with digital mammography alone (RR 1.13, 95% CI 0.96 to 1.32, Table 1). However, due to serious inconsistencies (DBT increased, decreased, and did not change the recall rates among different studies) the certainty of evidence was low. Due to very serious inconsistencies among retrospective cohort studies there was no clear effect of DBT on this outcome.

#### Positive Predictive Value (PPV) and False Positive Recalls Rate (Fig. 10 and Fig. 11, Supplementary File)

Based on RCT and accuracy studies, the effects of DBT plus digital mammography on false positive recalls and in the PPV for breast cancer were not different between the groups, however the quality of evidence was low due to imprecision and inconsistencies in the meta-analyses. The same occurred with the retrospective cohort studies.

### DBT plus synthetic mammography versus digital mammography alone

#### Breast Cancer Detection Rate (Fig. 12 to Fig. 14, Supplementary File)

With a moderate quality of evidence, and with 1,000 women screened, DBT plus synthetic mammography increased the overall and invasive breast cancer rates by 2 (RR 1.38, 95% CI 1.24 to 1.54 and RR 1.37, 95% CI 1.22 to 1.55, respectively, Table 1). The ductal breast cancer rates were marginally higher for DBT, but this difference was not statistically significant (RR 1.41, 95% CI 0.94 to 2.11).

#### Recall Rate (Fig. 15, Supplementary File)

DBT plus synthetic mammography results in no differences in recall rates (RR 1.08, 95% CI 0.92 to 1.26, Table 1). However, due to serious inconsistencies (recall rates increased, decreased, and did not change among the studies) the quality of evidence was low.

#### Positive Predictive Value and False Positive Recalls (Fig. 16 and Fig. 17, Supplementary File)

The effects of DBT plus synthetic mammography on false positive recalls for breast cancer were not different between the groups. However, the quality of evidence was low due to imprecision and inconsistencies in the meta-analyses,

Conversely, regarding patients recalled for additional assessment, DBT plus synthetic mammography resulted in little increase in the positive predictive value for breast cancer (RR 1.26, 95% CI 1.09 to 1.46), but due to serious imprecisions the quality of evidence was low.

### Additional analysis

The assessment of the accuracy of DBT (with digital or synthetic mammography versus digital mammography alone) could not be verified from the 2×2 contingency table data because it was impossible to confirm true and false negatives in all studies included.

None of the included studies evaluated overall or breast cancer mortalities or adverse events associated with DBT plus digital or synthetic mammography.

Regarding false negative rates, STORM (DBT plus digital mammography) was the only study that evaluated this outcome. In this accuracy study, the authors estimated the interval cancer rate at two-year follow-up and compared this result with a concurrent group of women who had attended the same screening services and received only digital mammography. The interval breast cancer rate in the STORM trial was not statistically different from that estimated amongst women screened with digital mammography (9/7292 screens versus 40/25,058 screens, respectively, RR 0,77, 95% CI 0.38 to 1.59), however the quality of evidence was low (wide confidence interval)^32^.

Only two studies presented the radiation dose per examination, Pattaccini *et al*. and Hofvind *et al*. (To-Be study), interventions which used DBT plus digital mammography and DBT plus synthetic mammography, respectively^24,25^. In the first study the median radiation dose per examination was 6.40 mGy (IQR, 5.68.–7.36 mGy) for DBT plus digital mammography and 4.84 mGy (IQR, 4.24–5.72 mGy) for digital mammography alone. In the second study, the mean radiation dose per examination was 2.96 mGy for DBT with synthetic mammography and 2.95 mGy for digital mammography alone. The remaining controlled studies only stated that the radiation dose levels of DBT plus digital mammography were approximately twice of those of digital mammography alone.

### Ongoing studies

There are two important clinical trials which currently in the recruitment phase. The first one is the Tomosynthesis Mammographic Imaging Screening Trial (TMIST)^72^. In this study, which is taking place in the United States, women aged 45 to 75 and attending a populational-based breast screening will be randomized to DBT or digital mammography. The researchers plan to enrol nearly 165,000 patients, and the primary outcome is the proportion of women diagnosed with advanced breast cancer at any time during a period of 4.5 years from randomization, including the period of active screening and a period of follow up after the last screen (time frame: 4.5 years after registration).

Another important ongoing clinical trial in this topic is called Digital Breast Tomosynthesis plus Synthesised Images versus Standard Full-Field Digital Mammography in Population-Based Screening (TOSYMA), which is being carried out in Germany. The authors aim to include 80,000 women aged 50 to 69 years who are attending their routine mammography screening programme^73^. The primary endpoints are the detection rate of invasive breast cancers during screening examinations and the cumulative incidence of interval cancers in the two years after a negative examination.

## Discussion

In order to present the best available evidence to help clinicians with decision making, we conducted a systematic review. The aim of this review was to compare the effect of DBT with digital mammography in over 45 year-old women attending a routine screening mammogram programme. Eighteen studies were included in this review. Our results show, with a moderate quality of evidence, that implementing DBT plus digital or synthetic mammography in population-based breast cancer screening increases overall breast cancer detection rates, as well as invasive breast cancer detection rates.

Although some studies have shown lower recall and false positives with DBT^6–8^, this was not confirmed in the present review. Our analyses did not find evidence for differences in recall rates between DBT (with digital or synthetic mammography) and digital mammography alone. However, due to the high heterogeneity between the results of the included studies, the quality of evidence was low.

In the context of breast cancer screening, a false negative finding can have devastating implications for the woman concerned, since a delay in cancer diagnosis can lead to an unfavourable evolution. In this review we did not find evidence of lower rates of false negatives with DBT. Conant *et al*., who compared the results of DBT with a historic cohort of digital mammography, evaluated the proportion of negative examinations in which cancer was diagnosed within 1 year. Results showed that the false-negative rates were slightly lower for DBT, but this difference was not statistically significant^74^. With the same study design Bahl *et al*. showed that the rate of interval cancers was similar with DM and DBT^75^.

We found 13 systematic reviews on this topic published in the literature^11–23^. Only one of these reviews had its protocol registered^15^, and all of their eligibility criteria were different from ours. Most reviews indicated that DBT with digital mammography was more effective, as it resulted in greater overall breast cancer detection and fewer false positives. However, none of them evaluated the quality of the evidence according to GRADE or included RCTs and prospective cohort studies in their analyses^24,47^.

Our systematic review had some limitations, the main one being related to the fact that none of the included studies evaluated the effects of DBT on improving breast cancer-related mortality, morbidity and quality of life. In a population-based cancer screening, besides the early cancer diagnosis, we sought to analyse the damage inflicted by these programs, including overdiagnosis and overtreatment at a very early stage of the disease. The Cochrane review estimated that for every 2,000 women invited to a mammography screening over a period of 10 years, one would have a long life, ten healthy women would suffer from overdiagnosis and overtreatment, and 200 women would suffer psychological damage due to false positive results^76^. Further, it has been estimated that breast cancer does not become symptomatic or health threatening in the lifetime of 1% to 10% of women with a positive diagnosis^77^. Additionally, it is estimated that overtreatment causes lifelong chronic pain in half of overdiagnosed women^76^.

## Conclusion

### Implications for clinical practice

Use of DBT with digital or synthetic mammography for women attending population-based breast cancer screenings increases the rates of overall and invasive breast cancer detection. There is no evidence, with high or moderate quality, showing that DBT, compared with digital mammography, decreases recall rates, as well as false positive and false negative rates.

### Implications for future research

Longitudinal studies are necessary to evaluate the effects of DBT on improving important patient outcomes (i.e. mortality, morbidity, test procedure complications, resource utilization, and quality of life).

## Supplementary information

Supplementary information 1.

Supplementary information 2.

## Data Availability

All data generated or analysed during this systematic review are included in this published article (and its Supplementary File).
