# Supplementary material for: Accuracy and Effectiveness of Mammography versus Mammography and Tomosynthesis for Population-Based Breast Cancer Screening: A Systematic Review and Meta-Analysis
Source: Sci Rep. 2020 May 14;10:7991. doi: 10.1038/s41598-020-64802-x (PMC7224282; doi:10.1038/s41598-020-64802-x)
Supplement: Supplementary file 2 — Supplementary information 2. [file 41598_2020_64802_MOESM2_ESM.pdf]

Accuracy and Effectiveness of Mammography versus Mammography and Tomosynthesis for Population-Based Breast Cancer Screening: A Systematic Review and Meta-Analysis

Rodrigo Rosa Giampietro[1], Marcos Vinicius Gama Cabral[1], Silvana Andrea Molina Lima [2,3], Silke Anna Theresa Weber[3,4], Vania dos Santos Nunes-Nogueira[1,3]

1. Department of Internal Medicine, Botucatu Medical School, São Paulo State University/UNESP, Sao Paulo, Brazil
2. Department of Nursing, Botucatu Medical School, São Paulo State University/UNESP, Sao Paulo, Brazil
3. Health Technology Assessment Nucleus, *Botucatu Medical School Clinical Hospital*, Sao Paulo, Brazil
4. Ophthalmology, Otorhinolaryngology and Head & Neck Surgery Department, Botucatu Medical School, São Paulo State University/UNESP, Sao Paulo, Brazil

Corresponding Author:

Vania dos Santos Nunes Nogueira

Departamento de Clínica Médica – FMB – UNESP

Avenida Professor Mário Rubens Guimarães Montenegro s/n, Bairro UNESP, Campus Botucatu, Botucatu-SP 18618-687, Brazil

Tel: (55 14) 3880 11 71

Fax: (55 14) 3880 16 67

E-mail: vania.nunes-nogueira@unesp.br

Supplementary File

Search strategies

2020/March/03

PUBMED

("digital"[All Fields] AND "breast"[All Fields] AND "tomosynthesis"[All Fields]) OR ("digital"[All Fields] AND "breast"[All Fields] AND "tomosyntheses"[All Fields]) OR ("digital breast tomosynthesis"[All Fields]) OR ("digital breast tomosyntheses"[All Fields]) OR ("breast"[All Fields] AND "tomosynthesis"[All Fields] AND "digital"[All Fields]) OR ("breast"[All Fields] AND "tomosyntheses"[All Fields] AND "digital"[All Fields])OR ("breast tomosynthesis, digital"[All Fields]) OR ("breast tomosyntheses, digital"[All Fields]) OR("3d"[All Fields] AND "mammography"[All Fields]) OR ("3d mammography"[All Fields]) OR ("3d"[All Fields] AND "mammography"[All Fields]) OR ("3d mammography"[All Fields]) OR ("3d"[All Fields] AND "mammographies"[All Fields]) OR ("3-d"[All Fields] AND "mammography"[All Fields]) OR ("3-d mammography"[All Fields]) OR ("3-d"[All Fields] AND "mammography"[All Fields]) OR ("3-d mammography"[All Fields]) OR ("3-d"[All Fields] AND "mammographies"[All Fields]) OR ("x ray breast tomosynthesis"[All Fields]) OR ("x ray breast tomosyntheses"[All Fields]) OR ("x- ray breast tomosynthesis"[All Fields]) OR ("x-ray breast tomosyntheses"[All Fields]) OR ("breast tomosynthesis, x- ray "[All Fields]) OR ("breast tomosyntheses, x- ray "[All Fields]) OR (" tomosynthesis"[All Fields]) OR (" tomosyntheses"[All Fields])

EMBASE

'digital breast tomosynthesis'/exp OR ' 3-D mammography' OR '3D mammography' OR 'Breast Tomosyntheses, Digital' OR 'Breast Tomosynthesis, Digital' OR 'Digital Breast Tomosynthese' OR '3D-Mammographies' OR 'X-ray Breast Tomosynthesis' OR 'Breast Tomosyntheses, X-ray' OR 'Breast Tomosynthesis, X-ray' OR 'X ray Breast Tomosynthesis' OR 'X-ray Breast Tomosyntheses' OR 'Tomosynthesis'

LILACS

MH:"Mammography" OR (digital breast tomosynthesis) OR (digital breast tomosyntheses) OR (breast tomosynthesis, digital) OR (breast tomosyntheses, digital) OR (3d mammography) OR (3-d

mammography) OR (3-d mammographies) OR (x ray breast tomosynthesis) OR (x ray breast tomosyntheses) OR (x- ray breast tomosynthesis) OR (x-ray breast tomosyntheses) OR (breast tomosynthesis, x- ray) OR (breast tomosyntheses, x- ray) OR (tomosynthesis) OR (tomosyntheses) OR (Mamografia tridimensional) OR (mamografia tomográfica) OR MH: E01.370.350.700.500

CENTRAL-COCHRANE

#1	tomosynthesis	49
#2	digital breast tomosynthesis	35
#3	breast tomosynthesis, digital 34	3
#4	3d mammography	16
#5	3-d mammography	4
#6	x ray breast tomosynthesis	12
#7	x- ray breast tomosynthesis	12
#8	Digital breast tomosynthesis	35
#9	MeSH descriptor: [Mammography] explode all trees	1045
#10	3D-Mammographies	0
#11	#1 or #2 or #3 or #4 or #5 or #6 or #7 or #8 or #9	1093

Table X. Characteristics of the included studies

Author/Year	Country	Study Design	Time of Study	Index Test	Comparison	DBT Manufacture	Number Radiologists Single/Double reading (sequentially / independently)	Inclusion/ Exclusion Criteria	Years Old (Included Women)	Differences between Family and Personal Background	Recall Criteria
Hofvind 2019 (To-Be)	Norway	RCT	Jan 2016 to Dec 2017	DBT and SM	DM alone	GE Healthcare (Chicago, IL, USA; SenoClaire 3D)	08 Double (independently)	Women who attend Breast Screen Norway with a complete screening exam and signed an informed consent/ No consent to participate, breast implants, breast cancer diagnosed before date of screening, metastatic melanoma, symptoms of breast cancer at the screening examination	50 to 69	-	A score of 2 or higher to one or both breasts (probably benign, intermediate suspicion; probably malignant; high suspicion of malignancy), there was a consensus meeting for further assessment
Pattacini 2018 (RETomo Study)	Northern Italy	RCT	Mar 2014 to Mar 2016	DBT and DM	DM alone	GE Senographe Essential digital systems (GE Healthcare, Buc, France)	02 Double	Women attending screening in one of three clinics equipped with DBT and who had already participated in at least one round of the Reggio Emilia screening program/Women with previous breast cancer, prior DBT, and ascertained genetic risk for breast cancer	45 to 70	Age was balanced (mean age was 56.2 years versus 56.3 years)	Proportion of women recalled for further assessment (after double reading and arbitration)
Houssami 2019	Maroondah BreastScreen (Australia)	Quasi-randomized pilot trial	Aug 2017 to Nov 2018	DBT and SM		Selenia; Dimensions 8000, with C-View 2D software, Hologic.	07 Double (independently)	Women who attend a routine biennial screening as part of the BreastScreen Victoria program/ Women who reported symptoms of breast cancer (sub analysis)	50 to 74	No significance difference for family and personal history of breast cancer	Need for further assessment in accordance with National Accreditation Standards of BreastScreen Australia
Hofvind 2018	Norway	Prospective Cohort	Feb 2014 to Jan 2016	DBT and SM	DM alone	Dimension; Hologic , (Beldfor, Mass)	16 Double (independently)	All Norwegian women aged 50–69 years to undergo biennial mammographic screening, with a stated time and place for the examination	50 to 69	-	BIRADS classification, consensus or arbitration meeting was held to determine the recall
Bernardi 2016 (STORM – 2)	Italy	DAT	May 2013 to May 2015	DBT, DM and SM	DM alone	Selenia Dimensions 2D/3D (Hologic)	07 Double (sequencially)	Asymptomatic women aged 49 years or older attending for biennial screening mammography provided to women at standard (population) risk for breast cancer	58 (53-63)	NA	A screen was classified as positive and the woman recalled to assessment for further investigations if recalled by either screen reader in either of the double-reading strategies based on recall at any screen-reading phase
Ciatto 2013 (STORM)	Ittaly	DAT	Aug 2011 to Aug 2012	DBT and DM	DM alone	Selenia Dimensions 2D/3D (Hologic)	08 Double (sequencially)	Asymptomatic women aged 49 years or older attending for biennial screening mammography provided to women at standard (population) risk for breast cancer	58 (48-71)	NA	Same as STORM – 2
Lang 2016 (MBTST)	Sweden	DAT	Jan 2010 to Dec 2012	DBT and DM	DM alone	Mammomat Inspiration (Siemens)	06 Single DBT, Double DM (independently)	All women aged 40–74 years are included in the Swedish breast cancer screening Programme. The exclusion criteria were pregnancy and women not speaking Swedish or English	56 (40-76)	NA	If one or both of the screening modalities was given a score of 3 or higher by one of the two readers (BI-RADS), it was referred for arbitration, where at least two readers re-evaluated the images and decided whether to recall the woman for further work-up.

Martin 2018	Spain	DAT	Jan 2015 to Dec 2016	DBT and SM	DM alone	Dimensions; Hologic; Bedford, A, USA and C view 2D software	05 Single	Women aged 50 to 69 years are invited to undergo routine biennial screening mammography	50 to 69	NA	Women were recalled for diagnostic work up if one or both readers consider the mammography as suspicious for malignancy
Skaane 2013	Norway	DAT	Nov 2010 to Dec 2011	DBT, DM and SM	DM alone	Dimension; Hologic , (Beldfor, Mass)	08 Double	Women (age range, 50–69 years) were invited to undergo routine, biennial, two-view (craniocaudal and mediolateral oblique) screening mammography during study period	50 a 69	NA	To receive a score ≥ 2 (Scale Used to Score Breasts and Cases), or after arbitration
Zervoudis 2013	Greece	DTA	2010 to 2012	DBT and DM	DM alone	Selenia BT device/FFDM (Hologic)	-	-	57 (34 a 85)	NA	Biopsy indication
Alsheik 2019	USA	Retrospective Cohort	Jun 2015 to Sep 2017	DBT and DM	DM alone	Hologic Selenia Dimensions / Siemens MAMMOMAT Inspiration	-	Women who underwent screening mammograms (DBT or DM) during the study period/Previous diagnosis of breast cancer, previous breast augmentation, or for lack of clinical information during the screening encounter	40 to 79	Women with dense breasts and higher calculated risk were more likely to be screened with DBT	BI-RADS 0, 3, 4 and 5; additional imaging needed
Cohen 2018	USA	Retrospective Cohort	Feb 2011 to Jun 2014	DBT and DM	DM alone	Selenia, Hologic	12 Single	Patients without at least 24 months of routine mammographic follow-up after the screening were excluded	40 to 70	The DBT group had a significantly higher personal and family history of breast cancer and a larger number of women with hyperdense breasts	Biopsy indication
Conant 2016	USA	Retrospective Cohort	2011 to 2014	DBT and DM	DM alone	Selenia Dimensions (Hologic)	14 -	All bilateral exams with an indication of screening and no other breast imaging within 3 months prior, among women 40–74 years of age with no known history of prior breast cancer	40 to 74 anos	DBT exams were more likely in women 40–49 years of age, among non-Hispanic black women, and among women with heterogeneously or extremely dense breasts	BI-RADS assessment category 0, 3, 4, or 5. Any biopsy occurring after screening, regardless of the BI-RADS assessment category of the exam.
Destounis 2014	USA	Retrospective Cohort	Jun 2011 to Dec 2011	DBT and DM	DM alone	Selenia Dimensions (Hologic)/Senographe Essential (GE Healthcare) /Fuji CRm, (Fujifilm)	06 Double	Women presenting for screening mammography at institution from June 2011 through December 2011	59 (36-92) / 59 (30-90)	DBT group had a significantly higher personal and family history of breast cancer and a larger number of women with hyperdense breasts.	BI-RADS scores entre 1 and 5, masses, microcalcifications, asymmetries, tissue overlap
Durand 2015	USA	Retrospective Cohort	Aug 2011 to Dec 2012	DBT and DM	DM alone	Selenia Dimensions (Hologic)/-Digital Mammographic Imager (Selenia, Hologic)	07 Single	Screening mammograms performed at their facility from August 1, 2011, to December 31, 2012. Women with breast implants or large breasts that required tiled images were excluded	<40 to >70	DBT exams were more likely in women 40–49 years of age, among non-Hispanic black women, and among women with heterogeneously or extremely dense breasts	Score BI-RADS 0, asymmetries, calcifications, masses
Giess 2017	USA	Retrospective Cohort	Oct 2012 to May 2015	DBT and DM	DM alone	Selenia Dimensions (Hologic)/Senographe DS. (GE Healthcare)	24 Single	Screening mammograms performed at study period	54,8 ± 10,3	The authors used a propensity score to counterbalance the factors associated with greater DBT (age <50 years, hyperdense breasts, calcifications or asymmetries in previous exams)	Score BI-RADS 0

Greenberg 2014	USA	Retrospective Cohort	Aug 2011 to Nov 2012	DBT and DM	DM alone	Selenia or Selenia Dimensions (Hologic)	14<br>Double	Screening mammograms performed at study period	59,5 (±10,31) /59,5 (± 11,44)	No difference between the groups regarding risk factors due to breast cancer	Score BI-RADS 0, 4 or 5
Powell 2017	USA	Retrospective Cohort	Jun 2012 to Aug 2014	DBT and DM	DM alone	Selenia e Selenia Dimensions (Hologic)	-	Women who attended breast cancer screening	>35 anos	The DBT group had more women with hyperdense breasts	Score BI-RADS 0

RCT: Randomized Clinical Trial. DTA: Diagnostic Accuracy Test Study. DBT: Digital Breast Tomosynthesis. DM: Digital Mammography. SM: Synthetic Mammography. NA: Not Applicable. – No Information Provided

Study or Subgroup	DBT + DM Events	DBT + DM Total	DM Events	DM Total	Weight	Risk Ratio M-H, Random, 95% CI
**1.1.1 Diagnostic Test Accuracy Studies**						
Bernardi 2016 (STORM-2)	82	9677	61	9677	17.4%	1.34 [0.97, 1.87]
Ciatto 2013 (STORM)	57	7292	39	7292	12.1%	1.46 [0.97, 2.19]
Lang 2016 (MBTST)	67	7500	47	7500	14.2%	1.43 [0.98, 2.07]
Skaane 2013 (OTST)	100	12621	77	12621	21.0%	1.30 [0.97, 1.75]
Zervoudis 2013	85	1615	79	1615	20.7%	1.08 [0.80, 1.45]
**Subtotal (95% CI)**		**38705**		**38705**	**85.3%**	**1.29 [1.11, 1.49]**
Total events	391		303			
Heterogeneity: Tau² = 0.00; Chi² = 2.12, df = 4 (P = 0.71); I² = 0%						
Test for overall effect: Z = 3.32 (P = 0.0009)						
**1.1.2 Randomized Clinical Trial**						
Pattacini 2018	83	9777	44	9783	14.7%	1.89 [1.31, 2.72]
**Subtotal (95% CI)**		**9777**		**9783**	**14.7%**	**1.89 [1.31, 2.72]**
Total events	83		44			
Heterogeneity: Not applicable						
Test for overall effect: Z = 3.42 (P = 0.0006)						
**Total (95% CI)**		**48482**		**48488**	**100.0%**	**1.36 [1.18, 1.58]**
Total events	474		347			
Heterogeneity: Tau² = 0.00; Chi² = 5.76, df = 5 (P = 0.33); I² = 13%						
Test for overall effect: Z = 4.10 (P < 0.0001)						
Test for subgroup differences: Chi² = 3.63, df = 1 (P = 0.06), I² = 72.5%						

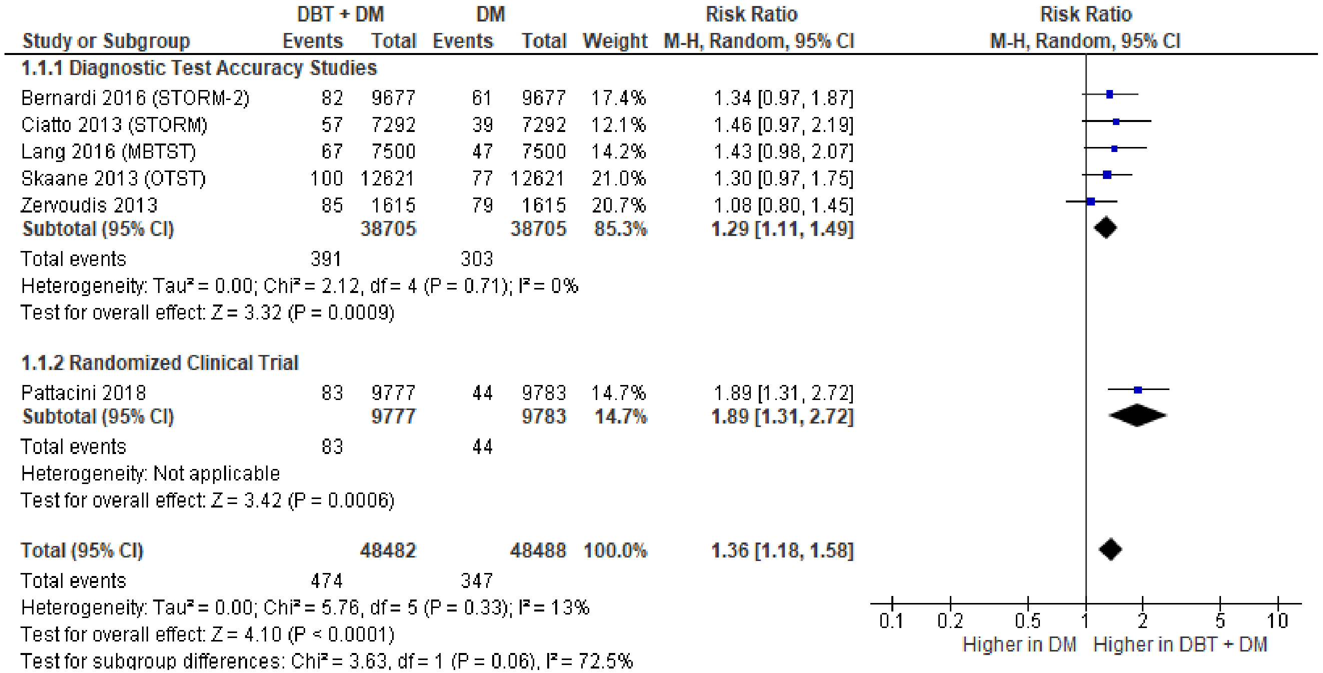

Fig. 3 Meta-analysis of the overall detection rate of breast cancer – digital breast tomosynthesis (DBT) and digital mammography (DM) versus digital mammography alone - diagnostic test accuracy studies and the randomized clinical trial.

Study or Subgroup	DBT + DM Events	DBT + DM Total	DM Events	DM Total	Weight	Risk Ratio M-H, Random, 95% CI
Alsheik 2019	497	95370	429	99660	43.8%	1.21 [1.06, 1.38]
Cohen 2018	28	71656	14	31414	2.0%	0.88 [0.46, 1.67]
Conant 2016	164	25268	551	113061	25.7%	1.33 [1.12, 1.58]
Destounis 2014	3	524	2	524	0.3%	1.50 [0.25, 8.94]
Durand 2015	51	8591	54	9364	5.7%	1.03 [0.70, 1.51]
Giess 2017	37	9817	26	14180	3.3%	2.06 [1.25, 3.39]
Greemberg 2014	130	20943	188	38674	16.2%	1.28 [1.02, 1.60]
Powell 2017	18	2304	54	10477	3.0%	1.52 [0.89, 2.58]
**Total (95% CI)**		**234473**		**317354**	**100.0%**	**1.26 [1.15, 1.38]**
Total events	928		1318			

Heterogeneity: $Tau^2 = 0.00$; $Chi^2 = 7.25$, df = 7 (P = 0.40); $I^2 = 3\%$

Test for overall effect: Z = 4.97 (P < 0.00001)

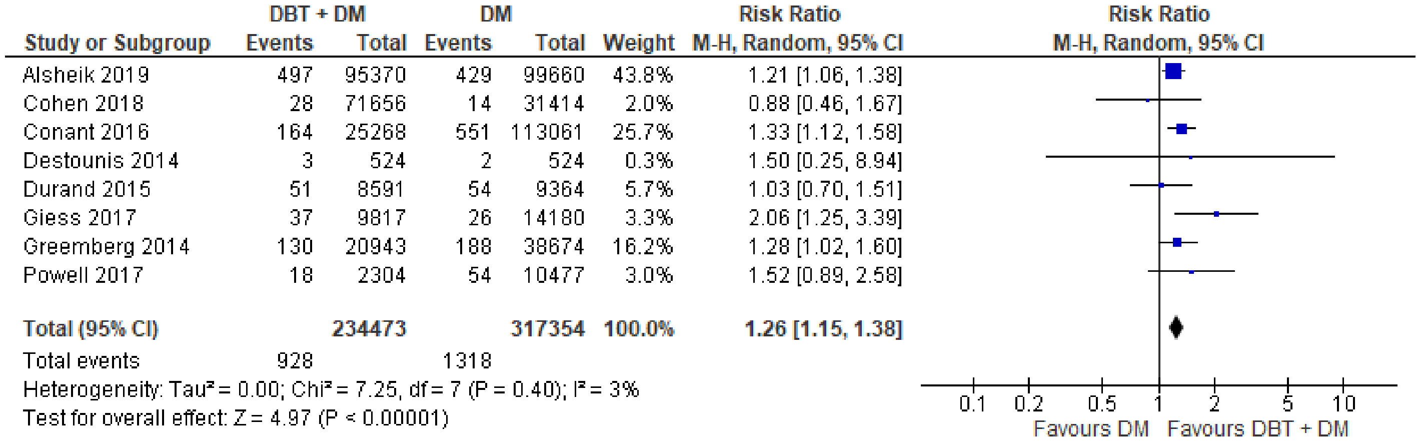

Fig. 4 Meta-analysis of the overall detection rate of breast cancer – digital breast tomosynthesis (DBT) and digital mammography (DM) versus digital mammography alone - **r**etrospective cohort studies

Study or Subgroup	DBT + DM Events	DBT + DM Total	DM alone Events	DM alone Total	Weight	Risk Ratio M-H, Random, 95% CI
**1.3.1 Invasive Breast Cancer - Diagnostic Test Accuracy Studies**						
Bernardi 2016 (STORM-2)	74	9677	46	9677	21.4%	1.61 [1.11, 2.32]
Ciatto 2013 (STORM)	52	7292	39	7292	16.8%	1.33 [0.88, 2.02]
Lang 2016 (MBTST)	58	7500	41	7500	18.1%	1.41 [0.95, 2.11]
Skaane 2013 (OTST)	81	12621	56	12621	24.9%	1.45 [1.03, 2.03]
**Subtotal (95% CI)**		**37090**		**37090**	**81.2%**	**1.46 [1.21, 1.76]**
Total events	265		182			
Heterogeneity: Tau² = 0.00; Chi² = 0.48, df = 3 (P = 0.92); I² = 0%						
Test for overall effect: Z = 3.91 (P < 0.0001)						
**1.3.2 Invasive Breast Cancer- Randomized Clinical Trial**						
Pattacini 2018	69	9777	39	9783	18.8%	1.77 [1.20, 2.62]
**Subtotal (95% CI)**		**9777**		**9783**	**18.8%**	**1.77 [1.20, 2.62]**
Total events	69		39			
Heterogeneity: Not applicable						
Test for overall effect: Z = 2.86 (P = 0.004)						
**1.3.3 Ductal Carcinoma in Situ - Diagnostic Test Accuracy Studies**						
Bernardi 2016 (STORM-2)	16	9677	15	9677	0.0%	1.07 [0.53, 2.16]
Ciatto 2013 (STORM)	7	7292	4	7292	0.0%	1.75 [0.51, 5.98]
Lang 2016 (MBTST)	7	7500	5	7500	0.0%	1.40 [0.44, 4.41]
Skaane 2013 (OTST)	20	12621	21	12621	0.0%	0.95 [0.52, 1.76]
**Subtotal (95% CI)**		**0**		**0**		**Not estimable**
Total events	0		0			
Heterogeneity: Not applicable						
Test for overall effect: Not applicable						
**1.3.4 Ductal Carcinoma in Situ - Randomized Clinical Trial**						
Pattacini 2018	14	9777	5	9783	0.0%	2.80 [1.01, 7.78]
**Subtotal (95% CI)**		**0**		**0**		**Not estimable**
Total events	0		0			
Heterogeneity: Not applicable						
Test for overall effect: Not applicable						
**Total (95% CI)**		**46867**		**46873**	**100.0%**	**1.51 [1.27, 1.79]**
Total events	334		221			
Heterogeneity: Tau² = 0.00; Chi² = 1.26, df = 4 (P = 0.87); I² = 0%						
Test for overall effect: Z = 4.76 (P < 0.00001)						
Test for subgroup differences: Chi² = 0.78, df = 1 (P = 0.38), I² = 0%						

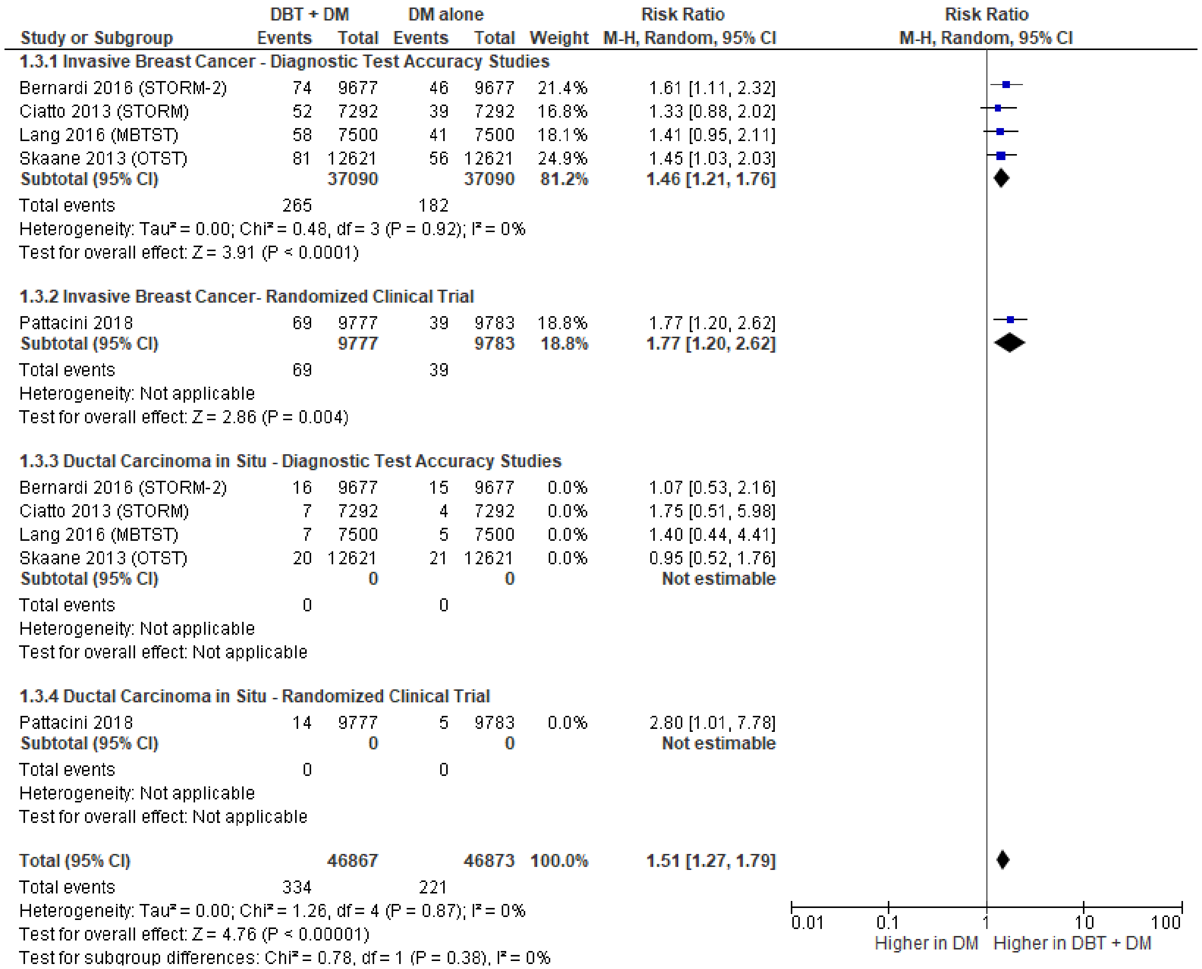

Fig. 5 Meta-analysis of the invasive breast cancer rate – digital breast tomosynthesis (DBT) and digital mammography (DM) versus digital mammography alone **-** diagnostic test accuracy studies and randomized clinical trial

Study or Subgroup	DBT + DM Events	DBT + DM Total	DM alone Events	DM alone Total	Weight	Risk Ratio M-H, Random, 95% CI
**1.3.1 Invasive Breast Cancer - Diagnostic Test Accuracy Studies**						
Bernardi 2016 (STORM-2)	74	9677	46	9677	0.0%	1.61 [1.11, 2.32]
Ciatto 2013 (STORM)	52	7292	39	7292	0.0%	1.33 [0.88, 2.02]
Lang 2016 (MBTST)	58	7500	41	7500	0.0%	1.41 [0.95, 2.11]
Skaane 2013 (OTST)	81	12621	56	12621	0.0%	1.45 [1.03, 2.03]
**Subtotal (95% CI)**		**0**		**0**		**Not estimable**
Total events	0		0			
Heterogeneity: Not applicable						
Test for overall effect: Not applicable						
**1.3.2 Invasive Breast Cancer- Randomized Clinical Trial**						
Pattacini 2018	69	9777	39	9783	0.0%	1.77 [1.20, 2.62]
**Subtotal (95% CI)**		**0**		**0**		**Not estimable**
Total events	0		0			
Heterogeneity: Not applicable						
Test for overall effect: Not applicable						
**1.3.3 Ductal Carcinoma in Situ - Diagnostic Test Accuracy Studies**						
Bernardi 2016 (STORM-2)	16	9677	15	9677	28.5%	1.07 [0.53, 2.16]
Ciatto 2013 (STORM)	7	7292	4	7292	9.4%	1.75 [0.51, 5.98]
Lang 2016 (MBTST)	7	7500	5	7500	10.7%	1.40 [0.44, 4.41]
Skaane 2013 (OTST)	20	12621	21	12621	37.8%	0.95 [0.52, 1.76]
**Subtotal (95% CI)**		**37090**		**37090**	**86.4%**	**1.11 [0.74, 1.66]**
Total events	50		45			
Heterogeneity: Tau² = 0.00; Chi² = 0.94, df = 3 (P = 0.82); I² = 0%						
Test for overall effect: Z = 0.50 (P = 0.62)						
**1.3.4 Ductal Carcinoma in Situ - Randomized Clinical Trial**						
Pattacini 2018	14	9777	5	9783	13.6%	2.80 [1.01, 7.78]
**Subtotal (95% CI)**		**9777**		**9783**	**13.6%**	**2.80 [1.01, 7.78]**
Total events	14		5			
Heterogeneity: Not applicable						
Test for overall effect: Z = 1.98 (P = 0.05)						
**Total (95% CI)**		**46867**		**46873**	**100.0%**	**1.26 [0.86, 1.83]**
Total events	64		50			
Heterogeneity: Tau² = 0.00; Chi² = 3.69, df = 4 (P = 0.45); I² = 0%						
Test for overall effect: Z = 1.19 (P = 0.23)						
Test for subgroup differences: Chi² = 2.74, df = 1 (P = 0.10), I² = 63.5%						

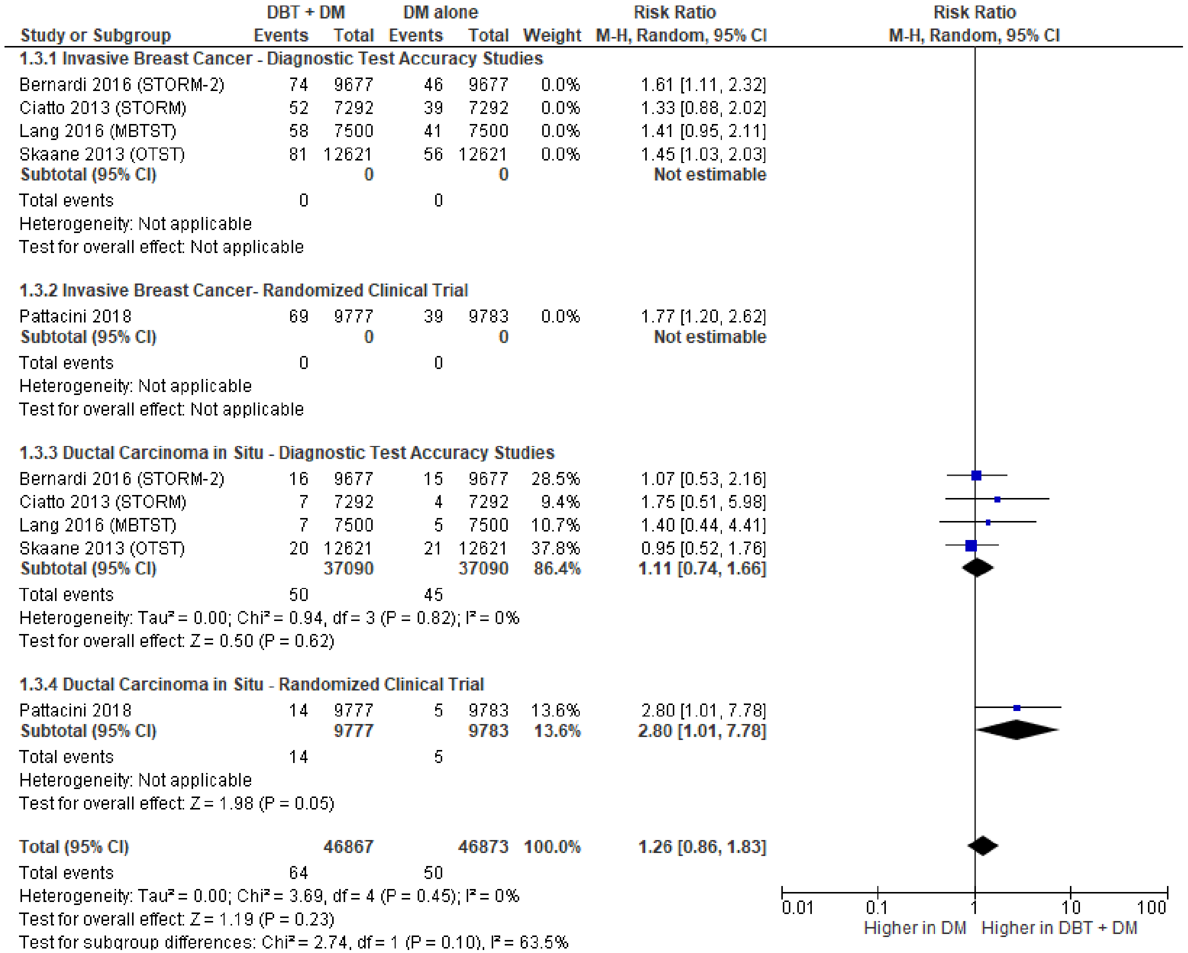

Fig. 6 Meta-analysis of the ductal carcinoma in situ rate – digital breast tomosynthesis (DBT) and digital mammography (DM) versus digital mammography alone - diagnostic test accuracy studies and randomized clinical trial

	DBT + DM		DM alone			Risk Ratio
**Study or Subgroup**	**Events**	**Total**	**Events**	**Total**	**Weight**	**M-H, Random, 95% CI**
Alsheik 2019	350	95370	328	99660	46.0%	1.12 [0.96, 1.30]
Conant 2016	119	25268	419	113061	26.7%	1.27 [1.04, 1.56]
Destounis 2014	1	524	1	524	0.2%	1.00 [0.06, 15.95]
Durand 2015	35	8591	35	9364	5.4%	1.09 [0.68, 1.74]
Giess 2017	28	9817	21	14180	3.7%	1.93 [1.09, 3.39]
Greemberg 2014	97	20943	122	38674	16.1%	1.47 [1.13, 1.92]
Powell 2017	8	2304	33	10477	2.0%	1.10 [0.51, 2.38]
**Total (95% CI)**		**162817**		**285940**	**100.0%**	**1.23 [1.10, 1.37]**
Total events	638		959			

Heterogeneity: Tau² = 0.00; Chi² = 6.20, df = 6 (P = 0.40); I² = 3%
Test for overall effect: Z = 3.71 (P = 0.0002)

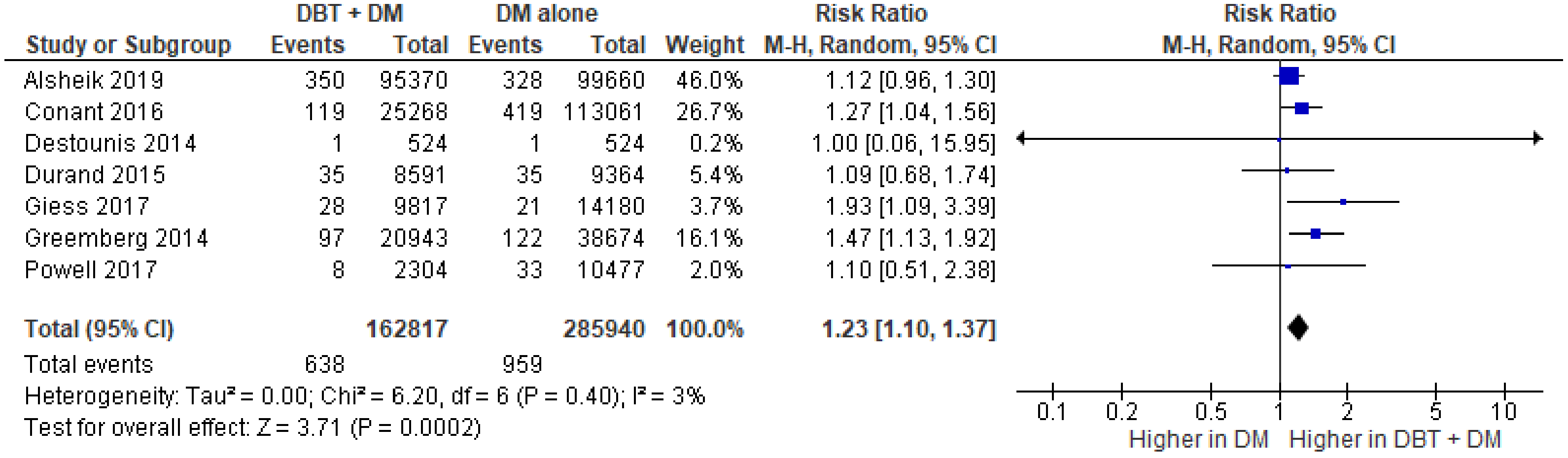

Fig. 7 Meta-analysis of the invasive detection rate of breast cancer – digital breast tomosynthesis (DBT) and digital mammography (DM) versus digital mammography alone - **r**etrospective cohort studies

Study or Subgroup	DBT + DM Events	DBT + DM Total	DM alone Events	DM alone Total	Weight	Risk Ratio M-H, Random, 95% CI
**1.5.1 Diagnostic Test Accuracy Studies**						
Bernardi 2016 (STORM-2)	463	9677	389	9677	18.4%	1.19 [1.04, 1.36]
Ciatto 2013 (STORM)	311	7292	361	7292	17.7%	0.86 [0.74, 1.00]
Lang 2016 (MBTST)	282	7500	197	7500	16.5%	1.43 [1.20, 1.71]
Skaane 2013 (OTST)	351	12621	265	12621	17.4%	1.32 [1.13, 1.55]
Zervoudis 2013	88	1615	86	1615	12.2%	1.02 [0.77, 1.37]
**Subtotal (95% CI)**		**38705**		**38705**	**82.2%**	**1.15 [0.95, 1.39]**
Total events	1495		1298			
Heterogeneity: Tau² = 0.04; Chi² = 24.32, df = 4 (P < 0.0001); I² = 84%						
Test for overall effect: Z = 1.48 (P = 0.14)						
**1.5.2 Randomized Clinical Trial**						
Pattacini 2018	344	9777	339	9783	17.8%	1.02 [0.88, 1.18]
**Subtotal (95% CI)**		**9777**		**9783**	**17.8%**	**1.02 [0.88, 1.18]**
Total events	344		339			
Heterogeneity: Not applicable						
Test for overall effect: Z = 0.20 (P = 0.84)						
**Total (95% CI)**		**48482**		**48488**	**100.0%**	**1.13 [0.96, 1.32]**
Total events	1839		1637			
Heterogeneity: Tau² = 0.03; Chi² = 26.51, df = 5 (P < 0.0001); I² = 81%						
Test for overall effect: Z = 1.50 (P = 0.13)						
Test for subgroup differences: Chi² = 1.07, df = 1 (P = 0.30), I² = 6.8%						

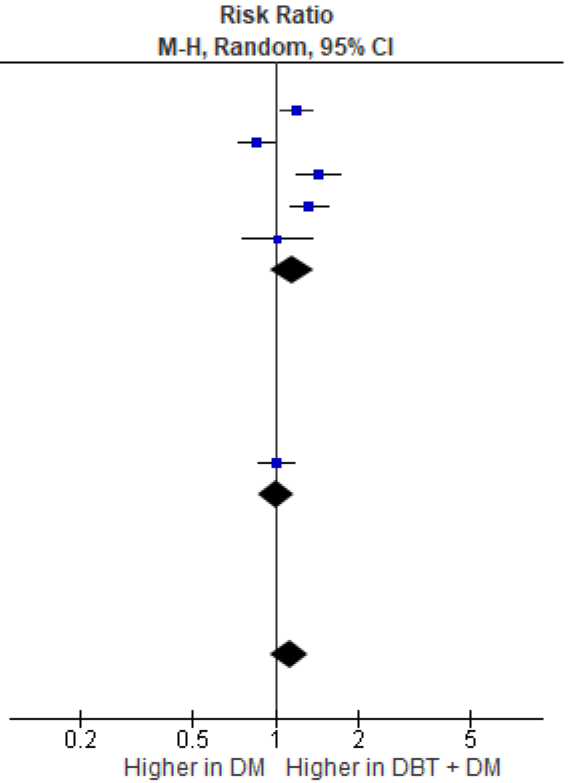

Fig. 8 Meta-Analysis of recall rates – digital breast tomosynthesis (DBT) and digital mammography (DM) versus digital mammography alone - diagnostic test accuracy studies and randomized clinical trial

Study or Subgroup	DBT+DM Events	DBT+DM Total	DM Events	DM Total	Weight	Risk Ratio M-H, Random, 95% CI
**1.10.1 Retrospective Cohort Studies**						
Alsheik 2019	17165	194437	14415	131292	13.0%	0.80 [0.79, 0.82]
Cohen 2018	1914	31414	5641	71656	12.9%	0.77 [0.74, 0.81]
Conant 2016	1099	25268	2547	113061	12.9%	1.93 [1.80, 2.07]
Destounis 2014	22	524	60	524	9.9%	0.37 [0.23, 0.59]
Durand 2015	671	8591	1154	9364	12.8%	0.63 [0.58, 0.69]
Giess 2017	2254	9817	1683	14180	12.9%	1.93 [1.83, 2.05]
Greenberg 2014	551	20943	835	38674	12.8%	1.22 [1.10, 1.36]
Powell 2017	319	2304	1694	10477	12.8%	0.86 [0.77, 0.96]
**Subtotal (95% CI)**		**293298**		**389228**	**100.0%**	**0.96 [0.70, 1.30]**
Total events	23995		28029			
Heterogeneity: Tau² = 0.19; Chi² = 1388.24, df = 7 (P < 0.00001); I² = 99%						
Test for overall effect: Z = 0.28 (P = 0.78)						
**Total (95% CI)**		**293298**		**389228**	**100.0%**	**0.96 [0.70, 1.30]**
Total events	23995		28029			
Heterogeneity: Tau² = 0.19; Chi² = 1388.24, df = 7 (P < 0.00001); I² = 99%						
Test for overall effect: Z = 0.28 (P = 0.78)						
Test for subgroup differences: Not applicable						

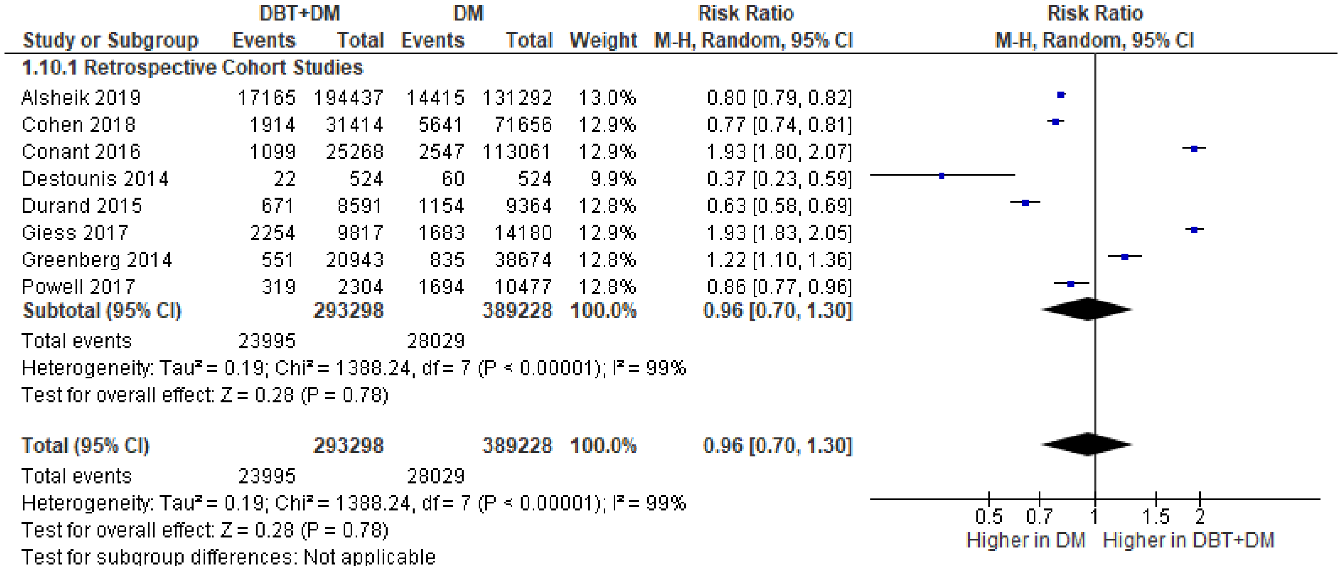

Fig. 9 Meta-Analysis of recall rates – digital breast tomosynthesis (DBT) and digital mammography (DM) versus digital mammography alone – retrospective cohort studies

	DBT + DM		DM alone			Risk Ratio
**Study or Subgroup**	**Events**	**Total**	**Events**	**Total**	**Weight**	**M-H, Random, 95% CI**
**1.6.1 Diagnostic Test Accuracy Studies**						
Bernardi 2016 (STORM-2)	381	9677	328	9677	20.3%	1.16 [1.00, 1.34]
Ciatto 2013 (STORM)	254	7292	322	7292	19.9%	0.79 [0.67, 0.93]
Lang 2016 (MBTST)	212	7500	150	7500	18.6%	1.41 [1.15, 1.74]
Skaane 2013 (OTST)	251	12621	188	12621	19.1%	1.34 [1.11, 1.61]
Zervoudis 2013	3	1615	7	1615	2.3%	0.43 [0.11, 1.65]
**Subtotal (95% CI)**		**38705**		**38705**	**80.2%**	**1.11 [0.86, 1.43]**
Total events	1101		995			
Heterogeneity: Tau² = 0.06; Chi² = 28.49, df = 4 (P < 0.00001); I² = 86%						
Test for overall effect: Z = 0.77 (P = 0.44)						
**1.6.2 Randomized Study**						
Pattacini 2018	261	9777	295	9783	19.8%	0.89 [0.75, 1.04]
**Subtotal (95% CI)**		**9777**		**9783**	**19.8%**	**0.89 [0.75, 1.04]**
Total events	261		295			
Heterogeneity: Not applicable						
Test for overall effect: Z = 1.45 (P = 0.15)						
**Total (95% CI)**		**48482**		**48488**	**100.0%**	**1.06 [0.85, 1.32]**
Total events	1362		1290			
Heterogeneity: Tau² = 0.05; Chi² = 34.03, df = 5 (P < 0.00001); I² = 85%						
Test for overall effect: Z = 0.54 (P = 0.59)						
Test for subgroup differences: Chi² = 2.06, df = 1 (P = 0.15), I² = 51.5%						

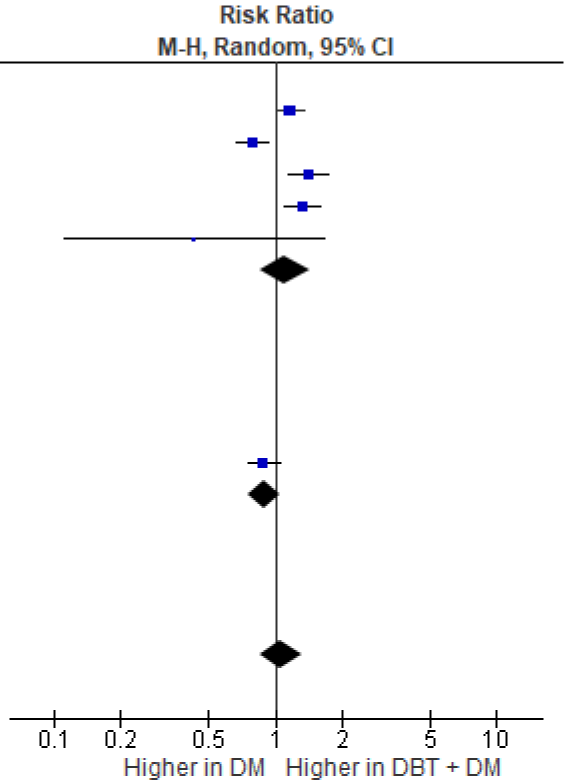

Fig. 10 Meta-Analysis of false positive for recall rates – digital breast tomosynthesis (DBT) and digital mammography (DM) versus digital mammography alone **-** diagnostic test accuracy studies and randomized clinical trial

Study or Subgroup	DBT + DM Events	DBT + DM Total	DM alone Events	DM alone Total	Weight	Risk Ratio M-H, Random, 95% CI
**1.7.1 Diagnostic Test Accuracy Studies**						
Bernardi 2016 (STORM-2)	82	463	61	389	16.1%	1.13 [0.83, 1.53]
Ciatto 2013 (STORM)	57	311	39	361	13.9%	1.70 [1.16, 2.48]
Lang 2016 (MBTST)	67	282	47	197	15.4%	1.00 [0.72, 1.38]
Skaane 2013 (OTST)	100	351	77	265	17.6%	0.98 [0.76, 1.26]
Zervoudis 2013	85	88	79	86	21.9%	1.05 [0.98, 1.13]
**Subtotal (95% CI)**		**1495**		**1298**	**84.8%**	**1.10 [0.94, 1.28]**
Total events	391		303			
Heterogeneity: Tau² = 0.01; Chi² = 8.29, df = 4 (P = 0.08); I² = 52%						
Test for overall effect: Z = 1.23 (P = 0.22)						
**1.7.2 Randomized Clinical Trial**						
Pattacini 2018	83	344	44	339	15.2%	1.86 [1.33, 2.59]
**Subtotal (95% CI)**		**344**		**339**	**15.2%**	**1.86 [1.33, 2.59]**
Total events	83		44			
Heterogeneity: Not applicable						
Test for overall effect: Z = 3.65 (P = 0.0003)						
**Total (95% CI)**		**1839**		**1637**	**100.0%**	**1.21 [0.97, 1.52]**
Total events	474		347			
Heterogeneity: Tau² = 0.06; Chi² = 25.96, df = 5 (P < 0.0001); I² = 81%						
Test for overall effect: Z = 1.66 (P = 0.10)						
Test for subgroup differences: Chi² = 7.82, df = 1 (P = 0.005), I² = 87.2%						

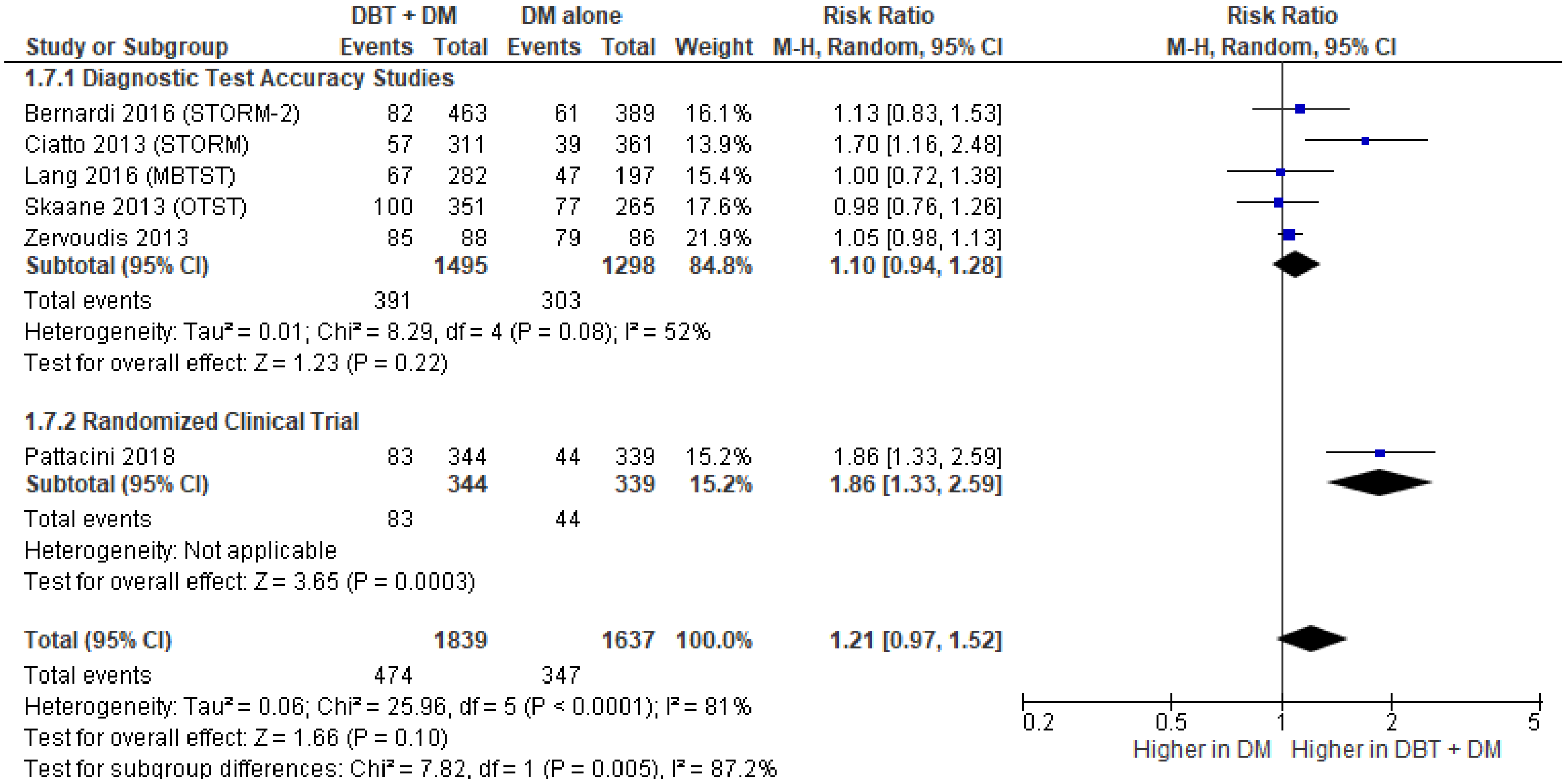

Fig. 11 Meta-Analysis of positive predictive value – digital breast tomosynthesis (DBT) and digital mammography (DM) versus digital mammography alone - diagnostic test accuracy studies and randomized clinical trial

Study or Subgroup	DBT + SM Events	DBT + SM Total	DM alone Events	DM alone Total	Weight	Risk Ratio M-H, Random, 95% CI
**1.8.1 Diagnostic Test Accuracy Studies**						
Bernardi 2016 (STORM-2)	85	9677	61	9677	10.2%	1.39 [1.00, 1.93]
Martin 2018	87	16067	66	16067	10.8%	1.32 [0.96, 1.81]
Skaane 2013 (OTST)	94	12621	77	12621	12.1%	1.22 [0.90, 1.65]
**Subtotal (95% CI)**		**38365**		**38365**	**33.1%**	**1.30 [1.09, 1.56]**
Total events	266		204			
Heterogeneity: Tau² = 0.00; Chi² = 0.35, df = 2 (P = 0.84); I² = 0%						
Test for overall effect: Z = 2.86 (P = 0.004)						
**1.8.2 Prospective Cohort**						
Hofvind 2018	348	37185	379	61742	48.4%	1.52 [1.32, 1.76]
**Subtotal (95% CI)**		**37185**		**61742**	**48.4%**	**1.52 [1.32, 1.76]**
Total events	348		379			
Heterogeneity: Not applicable						
Test for overall effect: Z = 5.70 (P < 0.00001)						
**1.8.3 RCT**						
Hofvind 2019 (To-Be)	95	14380	87	14369	13.0%	1.09 [0.82, 1.46]
Houssami 2019	45	4638	32	4883	5.4%	1.48 [0.94, 2.33]
**Subtotal (95% CI)**		**19018**		**19252**	**18.4%**	**1.21 [0.91, 1.60]**
Total events	140		119			
Heterogeneity: Tau² = 0.01; Chi² = 1.24, df = 1 (P = 0.26); I² = 20%						
Test for overall effect: Z = 1.31 (P = 0.19)						
**Total (95% CI)**		**94568**		**119359**	**100.0%**	**1.38 [1.24, 1.54]**
Total events	754		702			
Heterogeneity: Tau² = 0.00; Chi² = 5.14, df = 5 (P = 0.40); I² = 3%						
Test for overall effect: Z = 6.01 (P < 0.00001)						
Test for subgroup differences: Chi² = 2.97, df = 2 (P = 0.23), I² = 32.8%						

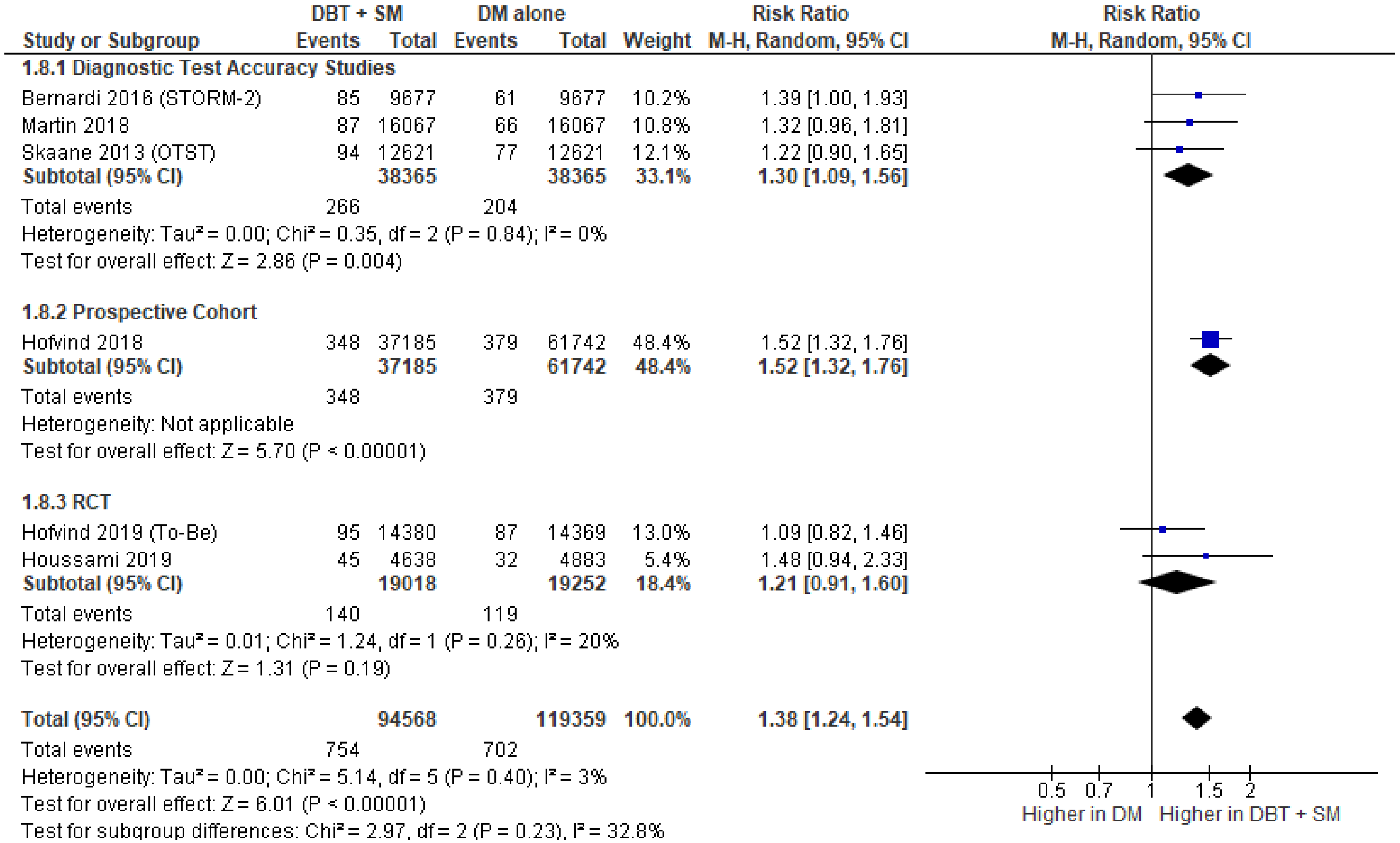

Fig. 12 Meta-analysis of the overall detection rate of breast cancer – digital breast tomosynthesis (DBT) and synthetic mammography (SM) versus digital mammography alone - diagnostic test accuracy studies, randomized clinical trial and prospective cohort study

Study or Subgroup	DBT + SM Events	DBT + SM Total	DM alone Events	DM alone Total	Weight	Risk Ratio M-H, Random, 95% CI
**1.12.1 Invasive Breast Carcinoma - Diagnostic Test Accuracy Studies**						
Bernardi 2016 (STORM-2)	74	9677	46	9677	10.6%	1.61 [1.11, 2.32]
Martin 2018	71	16067	57	16067	11.8%	1.25 [0.88, 1.76]
**Subtotal (95% CI)**		**25744**		**25744**	**22.5%**	**1.41 [1.09, 1.81]**
Total events	145		103			
Heterogeneity: Tau² = 0.00; Chi² = 0.98, df = 1 (P = 0.32); I² = 0%						
Test for overall effect: Z = 2.65 (P = 0.008)						
**1.12.2 Invasive Breast Carcinoma - Prospective Cohort Study**						
Hofvind 2018	283	37185	329	61742	57.0%	1.43 [1.22, 1.67]
**Subtotal (95% CI)**		**37185**		**61742**	**57.0%**	**1.43 [1.22, 1.67]**
Total events	283		329			
Heterogeneity: Not applicable						
Test for overall effect: Z = 4.41 (P < 0.0001)						
**1.12.3 Invasive Breast Carcinoma - RCT**						
Hofvind 2019 (To-Be)	80	14380	71	14369	14.1%	1.13 [0.82, 1.55]
Houssami 2019	40	5018	30	5166	6.4%	1.37 [0.86, 2.20]
**Subtotal (95% CI)**		**19398**		**19535**	**20.5%**	**1.20 [0.92, 1.56]**
Total events	120		101			
Heterogeneity: Tau² = 0.00; Chi² = 0.47, df = 1 (P = 0.50); I² = 0%						
Test for overall effect: Z = 1.34 (P = 0.18)						
**Total (95% CI)**		**82327**		**107021**	**100.0%**	**1.37 [1.22, 1.55]**
Total events	548		533			
Heterogeneity: Tau² = 0.00; Chi² = 2.74, df = 4 (P = 0.60); I² = 0%						
Test for overall effect: Z = 5.19 (P < 0.00001)						
Test for subgroup differences: Chi² = 1.30, df = 2 (P = 0.52), I² = 0%						

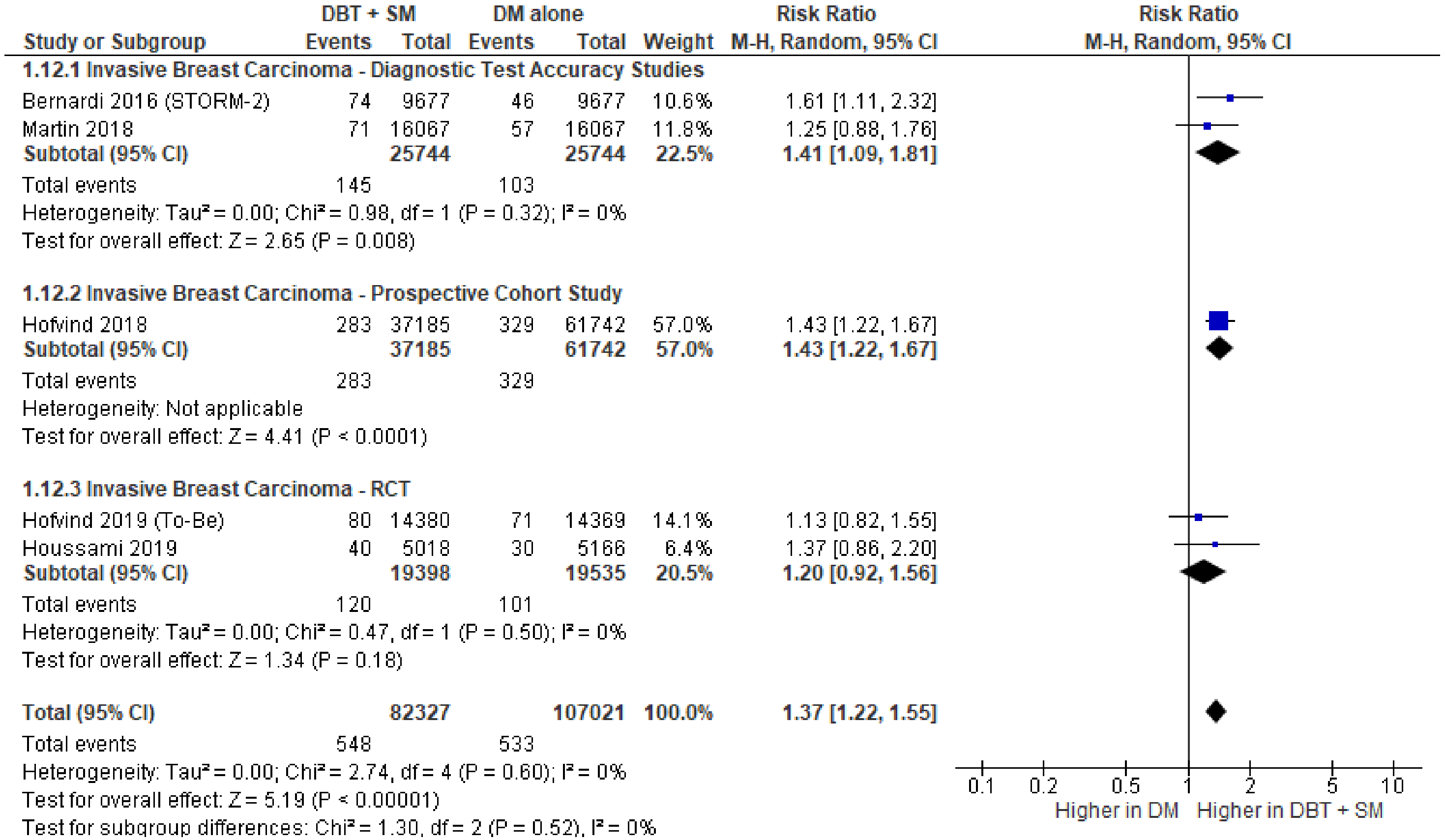

Fig. 13 Meta-analysis of the characteristics of breast cancer detection (invasive breast cancer)– digital breast tomosynthesis and synthetic mammography (SM) versus digital mammography alone - diagnostic test accuracy studies, randomized clinical trial and prospective cohort study

	DBT + SM		DM alone			Risk Ratio
**Study or Subgroup**	**Events**	**Total**	**Events**	**Total**	**Weight**	**M-H, Random, 95% CI**
**1.13.1 Ductal Carcinoma in Situ - Diagnostic Test Accuracy Studies**						
Bernardi 2016 (STORM-2)	16	9677	15	9677	18.7%	1.07 [0.53, 2.16]
Martin 2018	21	16067	19	16067	21.4%	1.11 [0.59, 2.05]
**Subtotal (95% CI)**		**25744**		**25744**	**40.1%**	**1.09 [0.68, 1.73]**
Total events	37		34			
Heterogeneity: Tau² = 0.00; Chi² = 0.01, df = 1 (P = 0.94); I² = 0%						
Test for overall effect: Z = 0.36 (P = 0.72)						
**1.13.2 Ductal Carcinoma in Situ - Prospective Cohort Study**						
Hofvind 2018	65	37185	50	61742	31.9%	2.16 [1.49, 3.12]
**Subtotal (95% CI)**		**37185**		**61742**	**31.9%**	**2.16 [1.49, 3.12]**
Total events	65		50			
Heterogeneity: Not applicable						
Test for overall effect: Z = 4.09 (P < 0.0001)						
**1.13.3 Ductal Carcinoma in Situ - RCT**						
Hofvind 2019 (To-Be)	15	14380	16	14369	18.7%	0.94 [0.46, 1.89]
Houssami 2019	9	5018	4	5166	9.2%	2.32 [0.71, 7.52]
**Subtotal (95% CI)**		**19398**		**19535**	**27.9%**	**1.30 [0.55, 3.04]**
Total events	24		20			
Heterogeneity: Tau² = 0.17; Chi² = 1.68, df = 1 (P = 0.20); I² = 40%						
Test for overall effect: Z = 0.60 (P = 0.55)						
**Total (95% CI)**		**82327**		**107021**	**100.0%**	**1.41 [0.94, 2.11]**
Total events	126		104			
Heterogeneity: Tau² = 0.10; Chi² = 7.74, df = 4 (P = 0.10); I² = 48%						
Test for overall effect: Z = 1.67 (P = 0.09)						
Test for subgroup differences: Chi² = 5.40, df = 2 (P = 0.07), I² = 63.0%						

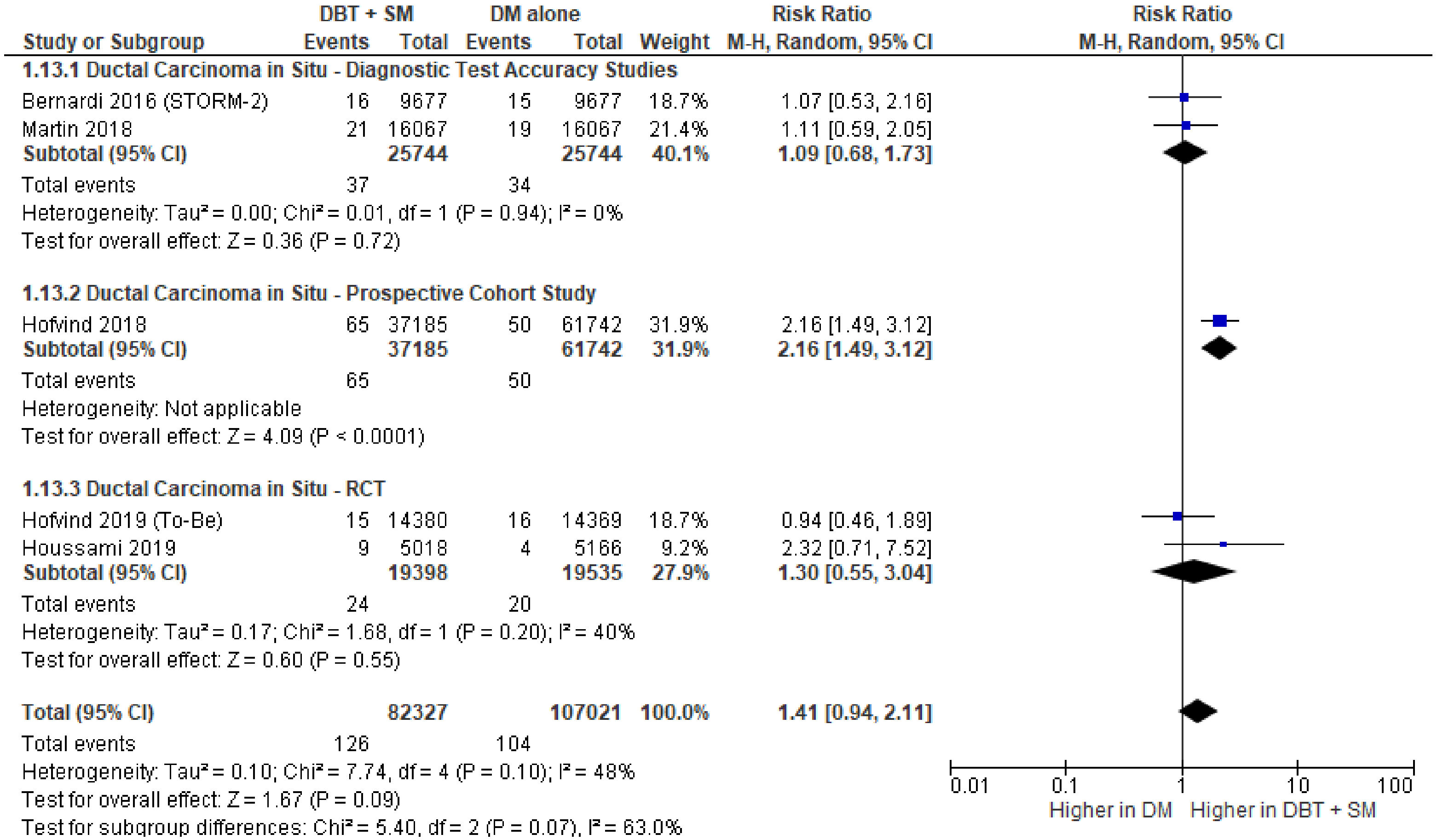

Fig. 14 Meta-analysis of the characteristics of breast cancer detection (ductal carcinoma in situ)– digital breast tomosynthesis and synthetic mammography (SM) versus digital mammography alone **-** Diagnostic test accuracy and randomized trial studies

Study or Subgroup	DBT + SM Events	DBT + SM Total	DM alone Events	DM alone Total	Weight	Risk Ratio M-H, Random, 95% CI
**1.9.1 Diagnostic Test Accuracy Studies**						
Bernardi 2016 (STORM-2)	512	9677	389	9677	17.0%	1.32 [1.16, 1.50]
Martin 2018	482	16067	499	16067	17.2%	0.97 [0.85, 1.09]
Skaane 2013 (OTST)	310	12621	265	12621	15.9%	1.17 [0.99, 1.38]
**Subtotal (95% CI)**		**38365**		**38365**	**50.1%**	**1.14 [0.94, 1.38]**
Total events	1304		1153			
Heterogeneity: Tau² = 0.02; Chi² = 11.79, df = 2 (P = 0.003); I² = 83%						
Test for overall effect: Z = 1.33 (P = 0.18)						
**1.9.2 Prospective Cohort**						
Hofvind 2018	1253	37185	2037	61742	18.6%	1.02 [0.95, 1.09]
**Subtotal (95% CI)**		**37185**		**61742**	**18.6%**	**1.02 [0.95, 1.09]**
Total events	1253		2037			
Heterogeneity: Not applicable						
Test for overall effect: Z = 0.60 (P = 0.55)						
**1.9.3 RCT**						
Hofvind 2019 (To-Be)	444	14390	571	14369	17.2%	0.78 [0.69, 0.88]
Houssami 2019	193	4638	144	4883	14.1%	1.41 [1.14, 1.74]
**Subtotal (95% CI)**		**19028**		**19252**	**31.3%**	**1.04 [0.58, 1.87]**
Total events	637		715			
Heterogeneity: Tau² = 0.17; Chi² = 22.92, df = 1 (P < 0.00001); I² = 96%						
Test for overall effect: Z = 0.13 (P = 0.90)						
**Total (95% CI)**		**94578**		**119359**	**100.0%**	**1.08 [0.92, 1.26]**
Total events	3194		3905			
Heterogeneity: Tau² = 0.03; Chi² = 46.50, df = 5 (P < 0.00001); I² = 89%						
Test for overall effect: Z = 0.95 (P = 0.34)						
Test for subgroup differences: Chi² = 1.11, df = 2 (P = 0.57), I² = 0%						

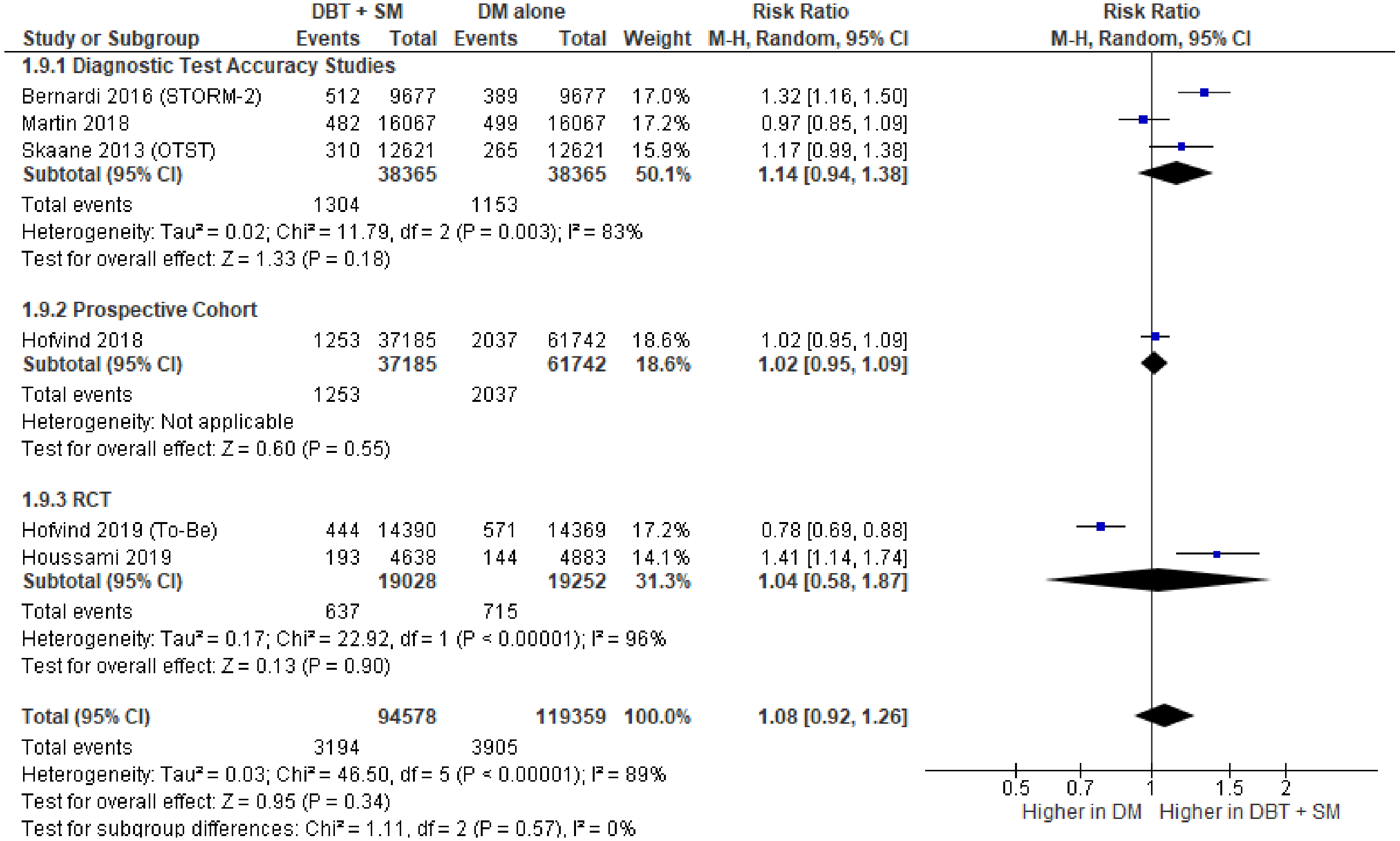

Fig. 15 Meta-Analysis of recall rates – digital breast tomosynthesis and synthetic mammography (SM) - Diagnostic test accuracy, randomized trial studies and prospective cohort study.

Study or Subgroup	DBT + SM Events	DBT + SM Total	DM alone Events	DM alone Total	Weight	Risk Ratio M-H, Random, 95% CI
**1.10.1 Accuracy Studies**						
Bernardi 2016 (STORM-2)	427	9677	328	9677	17.1%	1.30 [1.13, 1.50]
Martin 2018	395	16067	433	16067	17.3%	0.91 [0.80, 1.04]
Skaane 2013 (OTST)	216	12621	188	12621	15.7%	1.15 [0.95, 1.39]
**Subtotal (95% CI)**		**38365**		**38365**	**50.1%**	**1.11 [0.88, 1.39]**
Total events	1038		949			
Heterogeneity: Tau² = 0.03; Chi² = 13.08, df = 2 (P = 0.001); I² = 85%						
Test for overall effect: Z = 0.87 (P = 0.38)						
**1.10.2 Prospective Cohort**						
Hofvind 2018	905	37185	1658	61742	18.4%	0.91 [0.84, 0.98]
**Subtotal (95% CI)**		**37185**		**61742**	**18.4%**	**0.91 [0.84, 0.98]**
Total events	905		1658			
Heterogeneity: Not applicable						
Test for overall effect: Z = 2.41 (P = 0.02)						
**1.10.3 RCT**						
Hofvind 2019 (To-Be)	349	14380	484	14369	17.3%	0.72 [0.63, 0.83]
Houssami 2019	148	4638	112	4883	14.2%	1.39 [1.09, 1.77]
**Subtotal (95% CI)**		**19018**		**19252**	**31.5%**	**0.99 [0.52, 1.89]**
Total events	497		596			
Heterogeneity: Tau² = 0.21; Chi² = 21.58, df = 1 (P < 0.00001); I² = 95%						
Test for overall effect: Z = 0.02 (P = 0.98)						
**Total (95% CI)**		**94568**		**119359**	**100.0%**	**1.02 [0.85, 1.23]**
Total events	2440		3203			
Heterogeneity: Tau² = 0.04; Chi² = 49.92, df = 5 (P < 0.00001); I² = 90%						
Test for overall effect: Z = 0.25 (P = 0.80)						
Test for subgroup differences: Chi² = 2.67, df = 2 (P = 0.26), I² = 25.2%						

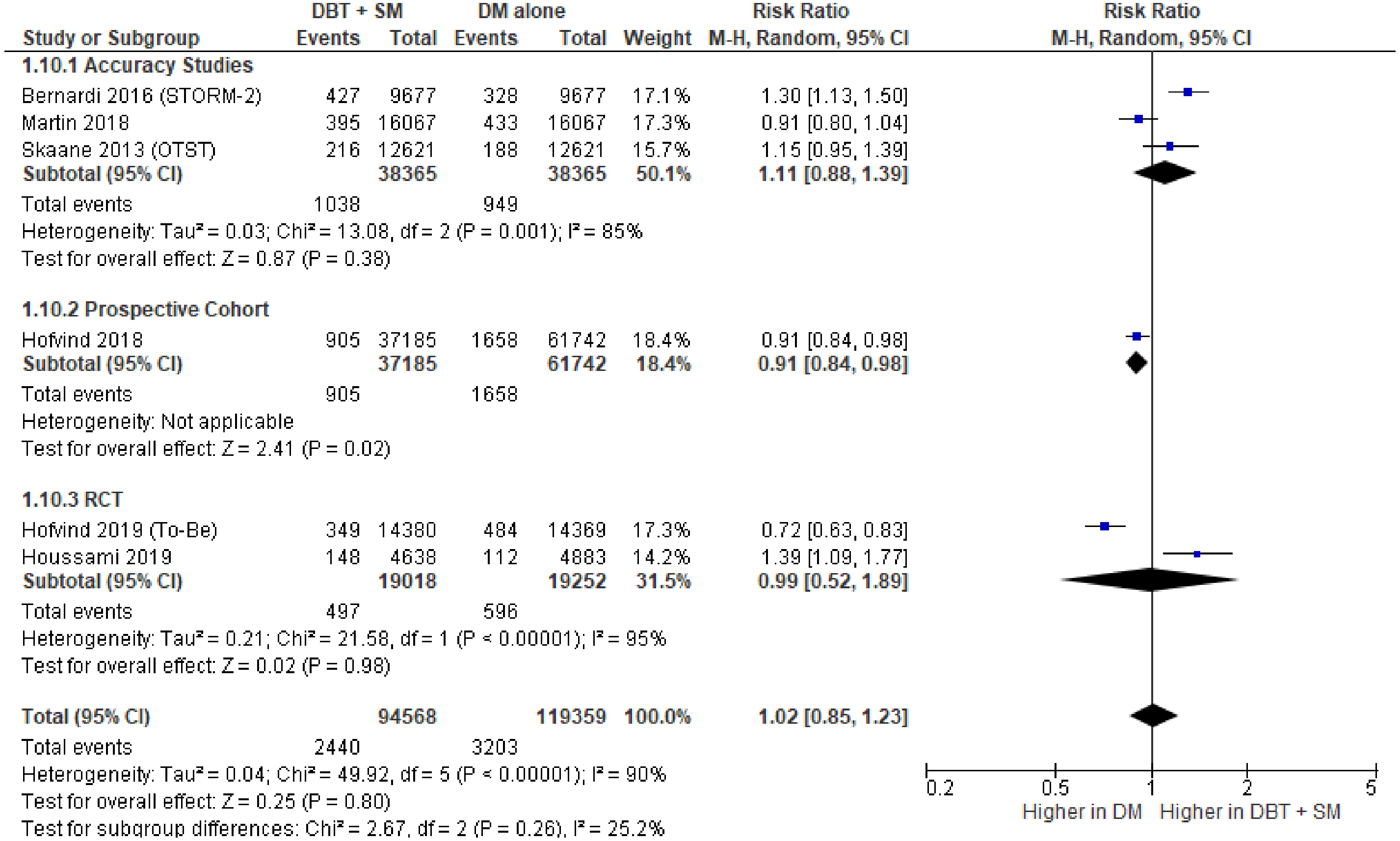

Fig. 16 Meta-Analysis of false positive recall – digital breast tomosynthesis and synthetic mammography (SM) versus - diagnostic test accuracy, randomized trial and prospective cohort study.

Study or Subgroup	DBT + SM Events	DBT + SM Total	DM alone Events	DM alone Total	Weight	Risk Ratio M-H, Random, 95% CI
**1.11.1 Diagnostic Test Accuracy Studies**						
Bernardi 2016 (STORM-2)	85	512	61	389	14.3%	1.06 [0.78, 1.43]
Martin 2018	87	482	66	499	14.7%	1.36 [1.02, 1.83]
Skaane 2013 (OTST)	94	310	77	265	17.2%	1.04 [0.81, 1.34]
**Subtotal (95% CI)**		**1304**		**1153**	**46.2%**	**1.14 [0.96, 1.34]**
Total events	266		204			
Heterogeneity: Tau² = 0.00; Chi² = 2.14, df = 2 (P = 0.34); I² = 7%						
Test for overall effect: Z = 1.50 (P = 0.13)						
**1.11.2 Prospective Cohort**						
Hofvind 2018	348	1253	379	2037	27.3%	1.49 [1.31, 1.70]
**Subtotal (95% CI)**		**1253**		**2037**	**27.3%**	**1.49 [1.31, 1.70]**
Total events	348		379			
Heterogeneity: Not applicable						
Test for overall effect: Z = 6.16 (P < 0.00001)						
**1.11.3 RCT**						
Hofvind 2019 (To-Be)	95	444	87	571	16.6%	1.40 [1.08, 1.83]
Houssami 2019	45	193	32	144	10.0%	1.05 [0.70, 1.56]
**Subtotal (95% CI)**		**637**		**715**	**26.5%**	**1.26 [0.96, 1.66]**
Total events	140		119			
Heterogeneity: Tau² = 0.01; Chi² = 1.43, df = 1 (P = 0.23); I² = 30%						
Test for overall effect: Z = 1.67 (P = 0.10)						
**Total (95% CI)**		**3194**		**3905**	**100.0%**	**1.26 [1.09, 1.46]**
Total events	754		702			
Heterogeneity: Tau² = 0.02; Chi² = 10.44, df = 5 (P = 0.06); I² = 52%						
Test for overall effect: Z = 3.03 (P = 0.002)						
Test for subgroup differences: Chi² = 6.62, df = 2 (P = 0.04), I² = 69.8%						

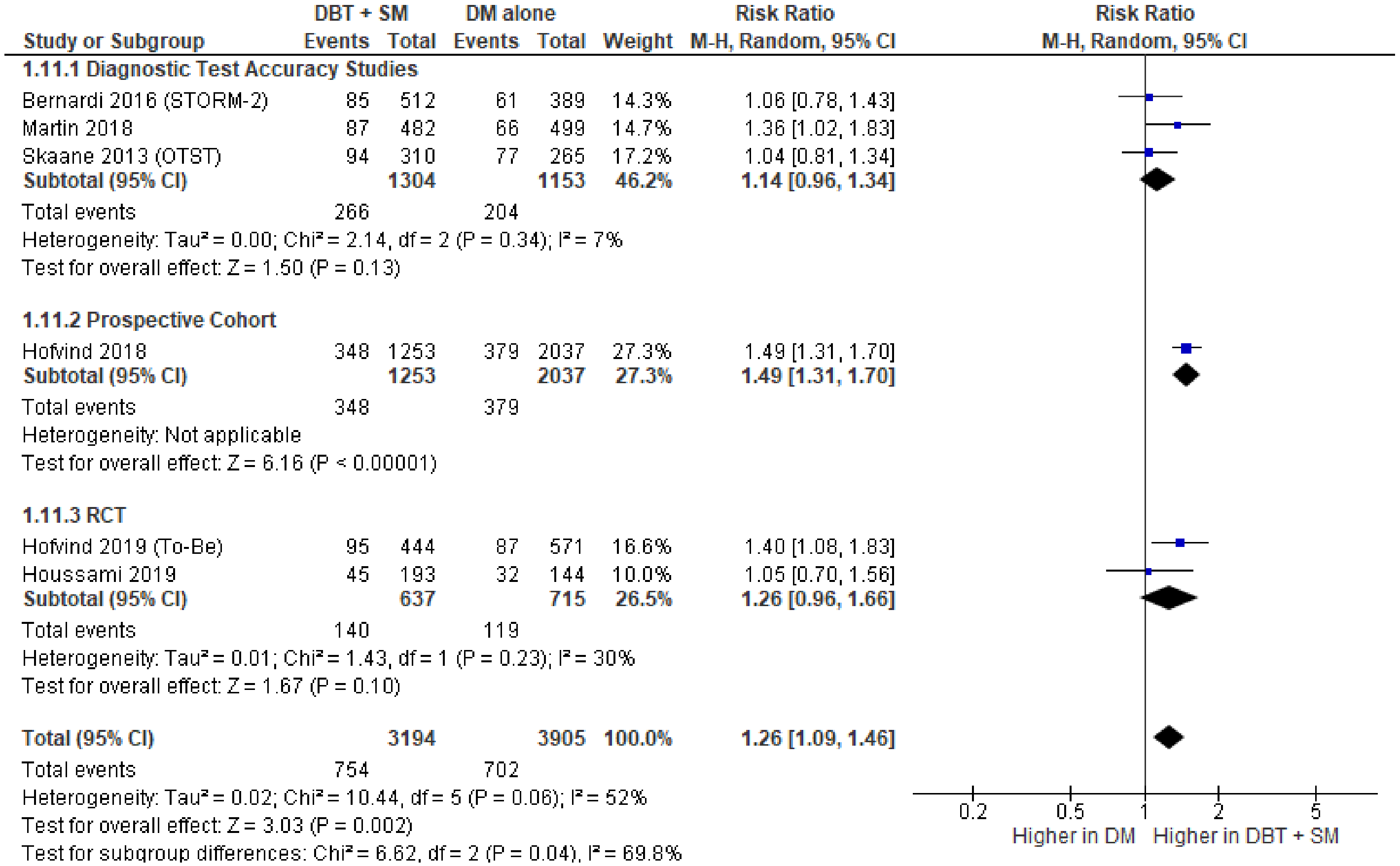

Fig. 17 Meta-Analysis of positive predictive value – digital breast tomosynthesis and synthetic mammography (SM) - diagnostic test accuracy, randomized trial and prospective cohort study.
